# Delineating the relationship between immune system aging and myogenesis in muscle repair

**DOI:** 10.1111/acel.13312

**Published:** 2021-01-28

**Authors:** Stephanie W. Tobin, Faisal J. Alibhai, Lukasz Wlodarek, Azadeh Yeganeh, Sean Millar, Jun Wu, Shu‐hong Li, Richard D. Weisel, Ren‐Ke Li

**Affiliations:** ^1^ Division of Cardiovascular Surgery Toronto General Research Institute University Health Network Toronto ON Canada

**Keywords:** aging, bone marrow transplant, inflammation, myogenesis, satellite cells

## Abstract

Recruited immune cells play a critical role in muscle repair, in part by interacting with local stem cell populations to regulate muscle regeneration. How aging affects their communication during myogenesis is unclear. Here, we investigate how aging impacts the cellular function of these two cell types after muscle injury during normal aging or after immune rejuvenation using a young to old (Y‐O) or old to old (O‐O) bone marrow (BM) transplant model. We found that skeletal muscle from old mice (20 months) exhibited elevated basal inflammation and possessed fewer satellite cells compared with young mice (3 months). After cardiotoxin muscle injury (CTX), old mice exhibited a blunted inflammatory response compared with young mice and enhanced M2 macrophage recruitment and *IL*‐*10* expression. Temporal immune and cytokine responses of old mice were partially restored to a young phenotype following reconstitution with young cells (Y‐O chimeras). Improved immune responses in Y‐O chimeras were associated with greater satellite cell proliferation compared with O‐O chimeras. To identify how immune cell aging affects myoblast function, conditioned media (CM) from activated young or old macrophages was applied to cultured C2C12 myoblasts. CM from young macrophages inhibited myogenesis while CM from old macrophages reduced proliferation. These functional differences coincided with age‐related differences in macrophage cytokine expression. Together, this study examines the infiltration and proliferation of immune cells and satellite cells after injury in the context of aging and, using BM chimeras, demonstrates that young immune cells retain cell autonomy in an old host to increase satellite cell proliferation.

## Graphical abstract

This study examined the infiltration and proliferation of immune cells and satellite cells in response to muscle injury during normal aging or after immune rejuvenation using a young to old (Y‐O) or old to old (O‐O) bone marrow transplant model. Old mice exhibited blunted inflammatory and myogenic responses compared to young mice with enhanced M2 macrophage recruitment. Immune rejuvenation in Y‐O chimeras restored the acute immune response in old mice and increased satellite cell proliferation.

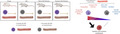

**Abbreviations**
BM: bone marrow
CM: conditioned media
CSA: cross‐sectional area
CTX: cardiotoxin
MSC: mesenchymal stem cell
O‐O: old to old
TA: tibialis anterior
Y‐O: young to old

## 1 INTRODUCTION

Satellite cells are tissue‐resident skeletal muscle stem cells which can proliferate and differentiate into mature myofibers. The satellite cell population is limited, and depletion of this key cell pool contributes to the decline in the regenerative capacity of skeletal muscle. Aging has a dramatic effect on satellite cell function (intrinsic functional changes) and cell number (Yin et al., 2013). For example, old muscle is subject to higher levels of muscle turnover; this leads to sustained activation of satellite cells and contributes to satellite cell depletion (Chakkalakal et al., 2012). Changes in satellite cell function also contribute to a decline in cell number as old cells exhibit higher levels of symmetric division, leading to fewer Pax7^+^ progenitor cells (Bernet et al., 2014). Moreover geriatric satellite cells may enter an irreversible state of quiescence (Sousa‐Victor et al., 2014), leading to reduced overall proliferative capacity. Satellite cells are also influenced by extrinsic factors such as factors in the circulation of old individuals, as demonstrated using heterochronic parabiosis and blood transfusion models (Conboy et al., 2003, 2005; Rebo et al., 2016). Mechanistically, these changes contribute to homeostatic and regenerative defects in skeletal muscle. Multiple approaches have been taken to improve satellite cell function in old individual in attempt to improve muscle regeneration and help maintain muscle function (Brack & Muñoz‐Cánoves, 2016). For example, several studies have shown that a youthful environment (by heterochronic parabiosis, blood transfusion, or muscle engraftment) can improve satellite cell function and subsequent muscle repair in old rodents (Carlson, 1989; Conboy et al., 2005; Rebo et al., 2016). In contrast, others have shown that more directed targeting approaches are needed to rejuvenate satellite cell function (Bernet et al., 2014; Cosgrove et al., 2014).

In addition to satellite cells, there are muscle‐resident cells such as macrophages, endothelial cells, and mesenchymal stem cells (MSCs). These cells help regulate the satellite cell microenvironment and modulate satellite cell maintenance. Muscle‐resident cells are largely distinguished by their cell surface markers, CD45^+^, CD31^+^, and Sca‐1^+^, which are generally associated with hematopoietic, endothelial, and mesenchymal stem cells, respectively (Liu et al., 2015). Muscle damage induces the robust recruitment of blood‐derived inflammatory cells, which play an active role in the repair process (Arnold et al., 2007; Summan et al., 2006; Sun et al., 2009). Immune cells are a critical component of the muscle repair response, as macrophage depletion alters satellite cell proliferation, prevents myogenesis, and results in fibrosis (Arnold et al., 2007; Summan et al., 2006; Sun et al., 2009). Old muscle suffers from deficiencies in macrophage function which lead to chronic inflammation, fibrosis (Wang et al., 2015), and a delayed inflammatory and myogenic response after injury (Shavlakadze et al., 2010; Smythe et al., 2008). Wang et al. showed that sarcopenia (age‐related muscle wasting) could be delayed when old mice received bone marrow transplant with young donor cells (Wang et al., 2019) but the populations, timing, and factors released by various inflammatory cells after heterochronic bone marrow chimerism in the context of muscle injury have not been adequately characterized.

The studies described above demonstrate that aging alters the skeletal muscle microenvironment, resulting in unfavorable tissue repair; however, using various approaches, rejuvenation of old satellite cell function is feasible. The bone marrow (BM) is reservoir of stem/progenitor cells, which we have shown to communicate with peripheral tissues to alter tissue repair (Li et al., 2013; Tobin et al., 2017; Wlodarek et al., 2020). Based on these studies, we investigated how BM aging affects the satellite cell population during rest and after acute injury in young vs old mice or after BM chimeras with young or old Sca‐1^+^ cells into old mice. Using flow cytometry analysis, cytokine, and muscle gene expression patterns, we profiled changes in BM‐derived immune cells, satellite cells, and MSCs in injured muscle during normal aging and using isochronic and heterochronic BM chimeras. We hypothesized that reconstituting old mice with young BM cells could rescue satellite cell function after acute muscle injury through differential recruitment of inflammatory cells and their respective cytokines.

## 2 RESULTS

### 2.1 Old muscle exhibits elevated inflammation at baseline

**FIGURE 1:**
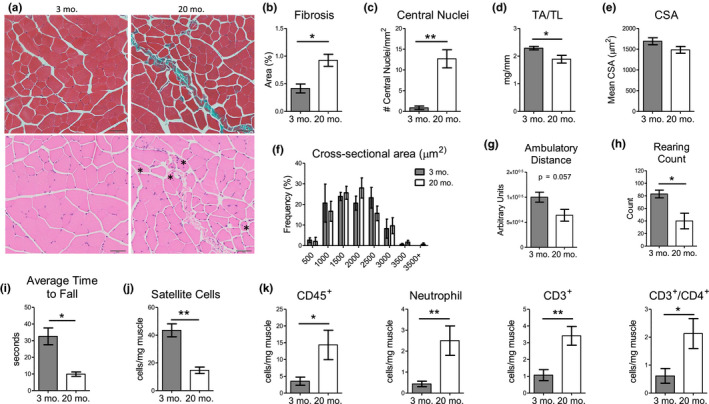
Age‐associated muscle deficiencies are coupled with inflammation. (a) Representative images of Masson's trichrome and H&E histological sections of the TA muscles of young (3‐month‐old) and old (>20‐month‐old) mice. Scale =50 µm. *Indicates centrally nucleated myofibers. (b) Quantification of intramuscular fibrosis based on trichrome staining of TA muscles from young and old mice (*n* = 3). (c) Quantification of central nuclei based on H&E staining of TA muscles from young and old mice (*n* = 3). (d) TA weight normalized to TL from young and old mice (*n* = 7). (e) Average CSA (µm^2^) of the TA muscle from young and old mice (*n* = 5). (f) Frequency plot of the CSA of the TA muscle from young and old mice grouped into 500 µm^2^ bins (*n* = 5). The open field test was used to assess ambulatory distance (g) and rearing count (h) (*n* = 4). (i) Assessment of the average time to fall using rotarod (*n* = 5). (j–k) Flow cytometry analysis of satellite cells (*n* = 4) or inflammatory cells (*n* = 5) from young and old mice. All data are presented as mean ± SEM. All analyses were done using an unpaired *t* test or two‐way ANOVA. *p < 0.05, **p < 0.01, ***p < 0.001. TA, Tibialis anterior; TL, Tibia length; CSA, Cross‐sectional area; H&E, Hematoxylin and eosin

We first characterized the physiological differences of skeletal muscles from young (3‐month‐old) and old (20‐month‐old) mice. With age, the degree of intramuscular fibrosis and muscle turnover (as indicated by centrally nucleated myofibers) was greater (Figure 1A‐C). However, the weight of the *Tibialis anterior* (TA) muscle was lower, as expected, in old mice (Figure 1D). There was no difference in the frequency distributions of myofiber sizes between these age groups, although the average cross‐sectional area (CSA) trended lower in older mice (Figure 1E‐F). We then performed several locomotive and behavioral tests as an indirect readout of muscle function: using the open field test we assessed ambulatory distance and rearing count and using the rotarod we measured the average time to fall. In all cases, old mice did significantly worse than young mice (Figure 1G‐I). These data highlight the deficits in muscle physiology and function that develop with age.

We next profiled the cellular differences between young and old muscles in terms of satellite cells, myeloid cells, and T cells. To identify satellite cells, we employed two gating methods using either VCAM‐1 or α7‐integrin. VCAM‐1 identifies both quiescent and activated satellite cells while α7‐integrin identifies a population of activated satellite cells (Liu et al., 2015; Maesner et al., 2016). Using both methods, we observed that old animals have fewer satellite cells (Figure 1J and Figure S1). Examination of CD45^+^ inflammatory cells revealed that they were more abundant in old muscle (Figure 1K). Changes in CD45^+^ cells were primarily driven by increased neutrophils and CD3^+^/CD4^+^ T cells, whereas the number of Ly6C^hi^ and Ly6C^lo^ monocytes and F4/80^+^ macrophages was not significantly affected by age, though some trended higher in old muscle (Figure S2). Examination of cytokine gene expression profiles in young and old muscle revealed that aging increased the expression of pro‐inflammatory cytokines *IL*‐*1β*, *IL*‐*12b*, and *IFNγ* but exhibited reduced expression of *IL*‐*23* and *IL*‐*10* (Figure S3).

### 2.2 Old muscle has a dampened immune response after injury

**FIGURE 2:**
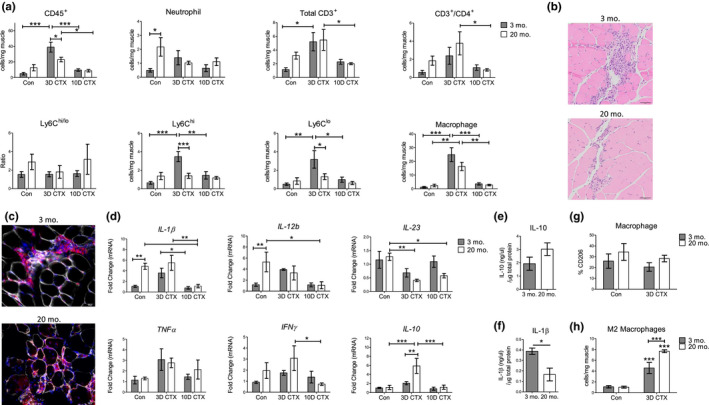
Young muscle produces a robust and transient inflammatory response that is impaired with age. (a) Flow cytometry analysis of immune cells in uninjured muscle and at 3‐ and 10 days post‐CTX injury in young (3‐month‐old) and old (20‐month‐old) mice. Uninjured (control) flow cytometry data are derived from Figure 1K and Figure S2. Control *n* = 6, 3D CTX *n* = 7, 10D CTX *n* = 5. Representative images of immune cell infiltration in young and old mice at 3 days post‐CTX using H&E (b) or immunofluorescent staining (c) for CD45 (red) and laminin (white). Scale = 20 µm. (d) Gene expression analysis of cytokines in the TA muscles of young and old mice at 3 and 10 days post‐CTX injury. The qRT‐PCR data are reported as relative to housekeeping gene, *Hprt* (*n* = 4). Protein quantification of IL‐10 (e) or IL‐1β (f) in young and old mice at 3D post‐CTX (*n* = 3–4). (g) The percentage and (h) cell number of CD206^+^ M2 macrophages at baseline or 3D post‐CTX. All data are presented as mean ± SEM. All analyses were done using an unpaired *t* test or two‐way ANOVA. *p < 0.05, **p < 0.01, ***p < 0.001. TA, Tibialis anterior; CTX, Cardiotoxin; Con, Control

To investigate how aging affects muscle repair, we profiled the cellular infiltration of immune cells and changes in cytokine expression in muscle after acute injury by cardiotoxin (CTX). We assessed the recruitment of neutrophils, macrophages, monocytes, and T cells at 3 and 10 days post‐injury. In young mice, overall CD45^+^ cells were significantly higher at 3 days post‐injury and returned to near baseline numbers by day 10 (Figure 2A). Although older muscle showed a similar trend in terms of total leukocyte number, the infiltration of CD45^+^ at 3 days post‐injury was significantly lower than in young muscle (Figure 2A). Representative images of inflammation at 3 days post‐injury are shown in Figure 2B (H&E stain) and Figure 2C (CD45 immunofluorescent stain) to show the magnitude and behavior of immune cell recruitment. It is evident from histological sections that the immune response is stronger in young muscle, correlating to flow cytometry data (Figure 2B). What is particularly striking in the immunofluorescent images is that necrotic myocytes overlapping with CD45 cells are still present in old muscle, likely to remove dead/dying cells, while in young muscle this process has been completed.

Recruitment of individual immune cell populations showed different trends. Neutrophils and T cells, which were more abundant in old muscle prior to injury, did not show any age‐related changes in recruitment post‐CTX. The number of neutrophils instead decreased after injury in old muscle. The most pronounced cellular difference was observed within the monocyte populations. Compared with young mice, old muscle had a negligible increase in Ly6C^hi^ and Ly6C^lo^ monocytes after injury (Figure 2A and representative flow cytometry images in Figure S4A). The total macrophage population followed a trend similar as monocyte infiltration, with fewer present in old muscle at 3D post‐CTX. Based on macrophage number normalized to muscle weight, this cell population accounts for the majority of CD45 cells at 3 days post‐CTX in both ages. As exemplified in representative flow cytometry images, macrophages rapidly infiltrate injured muscle of young and old mice at 3 days post‐CTX and largely disappear by 10 days post‐CTX (Figure S4B).

We next assessed the expression of 6 cytokines at baseline and 3 and 10 days post‐CTX (Figure 2D). In young muscle, following injury, the general trend of *IL*‐*1β* and *IL*‐*12b* was an upregulation at 3 days post‐CTX and a return to baseline levels by day 10. *IL*‐*12a* and *IL*‐*23* tended to be lower in old mice at 3 and 10 days post‐CTX. *IL*‐*10* was significantly higher in old muscle at 3 days post‐injury compared with young muscle, and *IFNγ* followed a similar trend. *TNFα* expression increased after injury but showed no age‐related differences in expression. Thus, although old muscle has higher pro‐inflammatory signals at baseline, the relative change in immune cells and pro‐inflammatory cytokines between uninjured and 3 days post‐CTX was stronger in young mice and impaired in old mice. We examined protein levels of IL‐10 and IL‐1β in 3‐ and 20‐month‐old mice at 3 days post‐CTX (Figure 2E‐F) and observed that the protein levels followed similar patterns of expression as mRNA expression: IL‐10 tended to be higher in old injured muscle, while IL‐1β was significantly upregulated in young injured muscle.

Using the M2 macrophage marker CD206, we assessed whether these differences could be attributed to any change in macrophage subpopulations, described here as M1 and M2 macrophage populations, respectively. The frequency of M2 macrophages was between 20 and 40% in both uninjured and injured muscle, across ages (Figure 2G). Following injury, the total number of M2 macrophages increased, and this population was significantly higher in old muscle (Figure 2H), indicating that aging affects the subpopulations of macrophages which populate the muscle following injury.

### 2.3 Heterochronic bone marrow chimerism of immune populations in uninjured skeletal muscle is not affected by age

**FIGURE 3:**
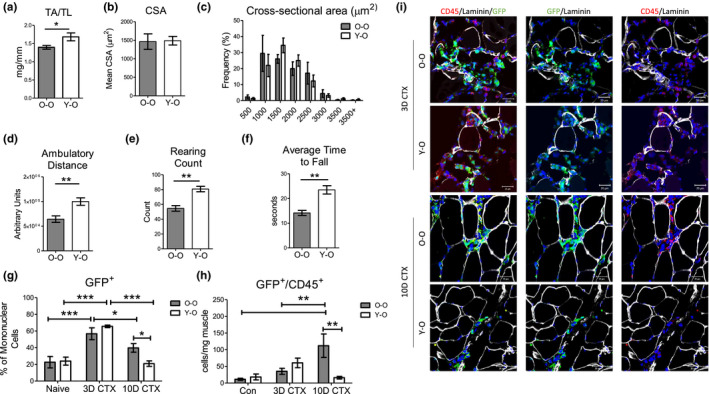
Analysis of muscle function and cellular profiles in old muscle after heterochronic bone marrow chimerism and tissue injury. (a) TA weight normalized to TL (*n* = 9). (b) Average CSA (µm^2^) of the TA muscle from Y‐O and O‐O mice (*n* = 5). (c) Frequency plot of the CSA of the TA muscle from Y‐O and O‐O mice grouped into 500 µm^2^ bins (*n* = 5). The open field test was used to assess (d) ambulatory distance and (e) rearing count (*n* = 5). (f) Rotarod was used to quantify the average time to fall (*n* = 4). (g) Percentage of GFP^+^ mononuclear cells in hindlimb muscle at baseline and post‐CTX. Con *n* = 4, 3D and 10D CTX *n* = 5. (h) Number of GFP^+^/CD45^+^ per mg muscle. Con *n* = 4, 3D CTX *n* = 5 10D CTX *n* = 5. (i) Representative images of staining for laminin (white), GFP (green), and CD45 (red) of TA cryosections of Y‐O and O‐O mice at 3 and 10 days post‐CTX. Scale = 20 µm. All data are presented as mean ± SEM. All analyses were done using an unpaired *t* test or two‐way ANOVA. In the case of two‐way ANOVA analysis, *p < 0.05, **p < 0.01, ***p < 0.001. TA, Tibialis anterior; TL, Tibia length; CTX, Cardiotoxin; BM, Bone marrow; Con, Control

To determine the extent to which inflammatory cell age affects myogenesis in old muscle, we used heterochronic BM transplant to generate chimeras. Sca‐1^+^ enriched BM cells from young or old GFP^+^ donor mice were transplanted into lethally irradiated old recipients as previously described (Li et al., 2017). Three months after BM transplant, hind limb muscle was collected for flow cytometry, gene expression and locomotive analysis. When reconstituted with young Sca‐1^+^ BM (Y‐O), the percentage of GFP^+^ cells present in muscle was similar to mice that received old Sca‐1^+^ BM (O‐O) (Figure S5A). Greater than 90% of all GFP^+^ mononuclear cells in muscle were CD45^+^, irrespective of donor age (data not shown). TA muscle mass was higher in Y‐O mice when normalized to tibia length but not body weight (Figure 3A and Figure S5B), which is lower in O‐O mice (data not shown). No significant differences in average cross‐sectional area (Figure 3B), intramuscular fibrosis, or centrally nucleated myofibers (Figure S5C‐E) were observed, though there was a slight shift in the frequency of myofibers to higher CSA in Y‐O mice (Figure 3C). With respect to behavioral and locomotive tests, young BM improved ambulatory distance, rearing count, and the average time to fall (Figure 3D‐F). Lastly, donor BM age did not affect the number of GFP^+^ muscle‐resident immune cell subpopulations at baseline (Figure S5E).

### 2.4 Y‐O mice exhibit improved immune cell recruitment following muscle injury

To determine the effect that BM rejuvenation has on the inflammatory response following muscle injury, reconstituted mice were injected with CTX and the percentage of GFP^+^ cells was assessed by flow cytometry 3 and 10 days later. Mononuclear cells in injured skeletal muscle were composed of 50–70% GFP^+^ cells (Figure 3G). This is dramatically higher than the 5–10% of GFP^+^ cells observed in uninjured muscle. Interestingly, O‐O chimeras had a higher proportion of GFP^+^ cells at 10 days post‐injury. The total number of GFP^+^/CD45^+^ cells normalized to muscle weight demonstrates the impact that age has on prolonged immune cell infiltration to muscle. GFP^+^/CD45^+^ cells peaked at 3 days post‐CTX and returned to baseline levels in Y‐O mice by day 10, while in O‐O mice this population was significantly above baseline levels at 10 days post‐CTX (Figure 3H). Representative flow cytometry images of GFP^+^/CD45^+^ cells are shown in Figure S6A. Figure 3I depicts immunofluorescent staining of the GFP^+^/CD45^+^ population between the Y‐O and O‐O groups at 3 and 10 days post‐injury. At 3 days post‐injury, GFP^+^/CD45^+^ cells are present within the site of muscle damage. At 10 days post‐injury, there was a higher amount of GFP^+^/CD45^+^ in mice that received old BM. These data demonstrate that in response in muscle injury, young immune cells retain their recruitment profiles in an old host, including a more robust immune response shortly after injury and faster resolution of the inflammatory phase.

### 2.5 Heterochronic bone marrow chimerism primarily influences the myeloid response to muscle injury in old mice

**FIGURE 4:**
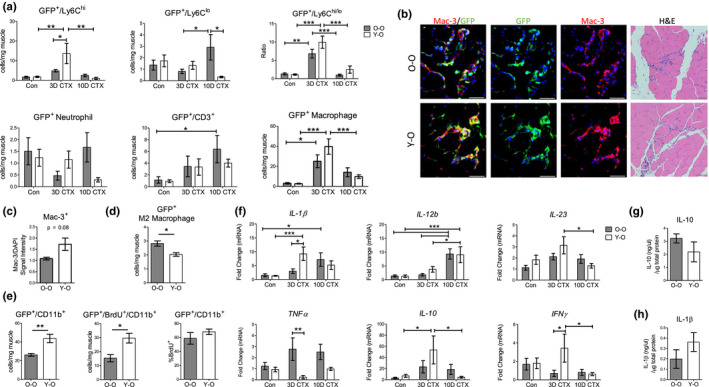
Young Sca‐1^+^ BM cells restore the timing of the myeloid response after acute muscle injury in old mice. (a) Flow cytometry analysis of GFP^+^ immune cells in injured muscle after heterochronic bone marrow chimerism. Control (Con) *n* = 4, 3D and 10D CTX *n* = 5. Control flow cytometry data are derived from Figure S5. (b) Immunofluorescent staining of macrophages in the TA muscle at 3 days post‐CTX. Dual staining for Mac‐3^+^ (red) and GFP^+^ (green) was done to identify differences in GFP^+^ macrophage recruitment in chimeras. H&E histological sections are included to the right. Scale =50 µm. (c) Immunofluorescent quantification of Mac‐3 signal intensity was normalized to DAPI signal intensity (*n* = 3). (d) GFP^+^/CD206^+^ M2 macrophage at 3D post‐CTX in Y‐O or O‐O cohorts (*n* = 4). (e) GFP^+^/CD11b^+^ cell proliferation at 3D post‐CTX assessed by BrdU incorporation and flow cytometry (*n* = 5). (f) Gene expression analysis of cytokines in the injured TA muscle at 3 and 10 days post‐CTX. The qRT‐PCR data are reported as relative to housekeeping gene, *Hprt* (*n* = 5–7). Protein quantification of (g) IL‐10 or (H) IL‐1β in O‐O and Y‐O chimeras at 3D post‐CTX (*n* = 3–5). All data are presented as mean ± SEM. All analyses were done using an unpaired *t* test or two‐way ANOVA. In the case of two‐way ANOVA analysis, *p < 0.05, **p < 0.01, ***p < 0.001. BM, Bone Marrow; TA, Tibialis Anterior; CTX, Cardiotoxin; Con, Control

We assessed cytokine gene expression and cellular recruitment of neutrophils, monocytes, macrophages, and T cells at each time point to better understand which cells were responsible for the age‐dependent differences we observed in CD45^+^ cell recruitment to injured muscle in O‐O or Y‐O mice. Monocytes were the main population affected by age, as at 3 days post‐CTX there were more GFP^+^/Ly6C^hi^ monocytes in Y‐O chimeras (Figure 4A). The late onset of immune cell infiltration in O‐O recipients at 10 days post‐CTX was derived from GFP^+^/Ly6C^lo^ monocytes and GFP^+^ T cells. GFP^+^ neutrophils were also more abundant at 10 days post‐CTX in O‐O chimeras compared with Y‐O mice (Figure 4A). Representative flow images show the accumulation of GFP^+^ macrophages, monocytes, and neutrophils (Figure S6B‐C). Flow cytometry analysis of GFP^+^ macrophages showed that this population trended higher in Y‐O mice after injury. A similar trend was seen using immunofluorescent cell staining for the general myeloid marker, Mac‐3 (Figure 4B‐C). Interestingly, there were fewer GFP^+^ M2 macrophage (CD206^+^) cells at 3 days post‐CTX between Y‐O and O‐O mice (Figure 4D). Examination of Bromodeoxyuridine (BrdU) incorporation in CD11b^+^ cells, a general myeloid cell marker, also demonstrated higher rates of myeloid cell proliferation at 3 days post‐injury, suggesting that young immune cells retain a higher proliferative capacity than old cells, even in an old environment (Figure 4E).

We next assessed the expression of six cytokines during this repair process: *IL*‐*1β*, *IL*‐*12b*, *IL*‐*23*, *TNFα*, *IFNγ*, and *IL*‐*10* (Figure 4F). Y‐O mice had higher expression of *IL*‐*1β* and *IFNγ* at 3 days post‐CTX compared with O‐O mice. Notably, *IL*‐*1β* expression increased in O‐O muscle much later at 10 days post‐CTX, consistent with impaired resolution of the pro‐inflammatory phase. *IL*‐*12b* expression increased in both Y‐O and O‐O at 10 days post‐CTX reflecting a delayed onset inflammation phenotype that coincided with T‐cell recruitment (Figure 4A). The only factor upregulated in the O‐O chimeras was *TNFα*, which was significantly upregulated at 3 days post‐injury. No age‐related change in *IL*‐*10* expression was observed.

Lastly, we examined IL‐10 and IL‐1β protein levels in O‐O and Y‐O muscles, 3 days after injury (Figure 4G‐H). IL‐10 protein levels trended lower, while IL‐1β protein levels trended higher, similar to what was observed in 3‐ and 20‐month‐old injured muscles (Figure 3E‐F); however, there were no significant differences between chimeras. These patterns paralleled the observations from our flow cytometry and mRNA data, which show that heterochronic bone marrow chimerism with young cells restores the acute inflammatory response after muscle injury.

### 2.6 Heterochronic bone marrow chimerism influences host cell proliferation but does not significantly improve myogenesis in response to acute injury

**FIGURE 5:**
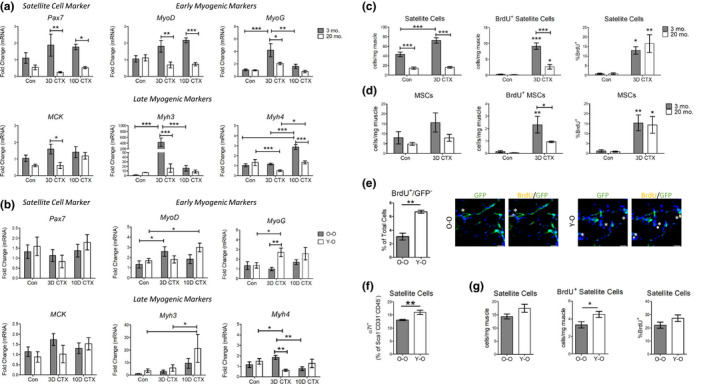
Satellite cell proliferation is impaired with aging and improved by heterochronic bone marrow chimerism. (a) Gene expression analysis of satellite cell and early and late myogenic markers in the TA muscle from young (3‐month‐old) or old (20‐month‐old) mice. The qRT‐PCR data are reported as relative to housekeeping gene, *Hprt* (*n* = 4). (b) Gene expression analysis of satellite cell and early and late myogenic markers in the TA muscle of CTX‐injured mice after heterochronic BM chimerism. Con *n* = 7, 3D *n* = 7, 10D CTX *n* = 6. (c) Satellite cell and (d) MSC proliferation in young or old mice assessed by flow cytometry (*n* = 4). (e) Immunofluorescence and quantification of BrdU^+^/GFP^−^ cells 3D post‐CTX in Y‐O and O‐O mice. The left panel quantifies the number of BrdU^+^/GFP^−^ cells per total number of cells per image, and the right panels show representative images of staining for GFP (Green) and BrdU (Yellow) of TA cryosections from Y‐O and O‐O mice 3D post‐CTX (*n* = 3). (f–g) Quantification of satellite cell proliferation assessed by flow cytometry in Y‐O and O‐O mice. (F) Percentage of GFP^−^ satellite cells in muscle 3D post‐CTX (*n* = 5). (g–h) Satellite cell number and proliferation (BrdU^+^) 3D post‐CTX (*n* = 5). All data are presented as mean ± SEM. All analyses were done using an unpaired *t* test or two‐way ANOVA. *p < 0.05, **p < 0.01, ***p < 0.001. BM, Bone Marrow; TA, Tibialis Anterior; CTX, Cardiotoxin; Con, Control; MSC, Mesenchymal Stem Cell

We next assessed the influence that BM age has on the myogenic response (Figure 5). First, we assessed how WT young or old muscles respond to injury and demonstrated that aging strongly influences myogenic capacity (Figure 5A). We grouped myogenic genes based on their function: *Pax7* is a satellite cell marker; Myogenin (*MyoG*) and *MyoD* are early myogenic markers representing commitment and differentiation of myoblasts; *MCK*, *Myh3*, and *Myh4* are late myogenic markers, representing maturation of the myofiber. After injury, *Myh3* is expressed in newly formed myofibers and is gradually replaced by *Myh4*. *Pax7*, and *MyoD* expression was significantly higher in young muscle at 3 and 10 days post‐injury compared with old muscle. In young muscle, *MyoG* expression was elevated at 3 days and returned to baseline levels by 10 days post‐CTX. Old muscle showed similar patterns of *MyoG* expression however this was significantly lower than in young muscle. In young muscle, late myogenic markers (*MCK* and *Myh3*) increased by 3 days post‐CTX while *Myh4* expression was not upregulated until 10 days post‐injury. Compared with young muscle, expression of *MCK* and *Myh3* was significantly lower in old muscle at 3 days post‐CTX. This trend extended to 10 days post‐CTX when *Myh4* expression was higher in young muscle. We indirectly evaluated muscle function at 7 days post‐CTX using the open field test and observed that young mice had more rearing counts and greater ambulatory distance than old mice (Figure S7A‐B). In Y‐O chimeras, these measurements trended higher than in O‐O mice (Figure S7C‐D).

CTX injury on Y‐O or O‐O chimeras resulted in some age‐related differences that were partially rescued by young BM. Notably, *MyoG* expression was higher at 3 days post‐CTX in Y‐O chimeras, potentially representing differences in satellite cell proliferation (Figure 5B). In contrast, *Myh4*, a late myogenic marker, was downregulated at 3 days post‐CTX in the Y‐O group. No significant changes were observed in *Pax7* or *MCK* expression but *MyoD* and *Myh3* expression trended higher in the Y‐O cohort at 10 days post‐injury.

**FIGURE 6:**
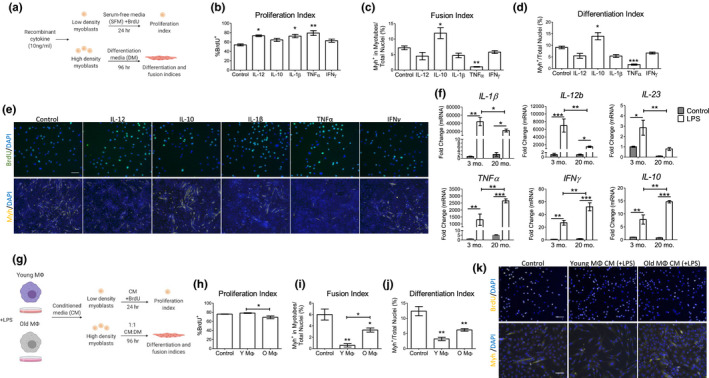
Age‐related changes in pro‐ and anti‐inflammatory cytokines alter myoblast proliferation and differentiation. (a–e) The effect of cytokines on C2C12 myoblast proliferation and differentiation. (a) Schematic depiction of C2C12 culture conditions used to assess the role of five cytokines on muscle cell proliferation and differentiation. (b) Proliferation index of cells following cytokine treatment using the number of BrdU^+^ nuclei (*n* = 3). (c–d) Differentiation and fusion index of C2C12 myoblasts after culture in differentiation medium supplemented with the indicated cytokine for 96 h (*n* = 3). (e) Representative images of BrdU^+^ (green) or Myh^+^ (yellow) cells used to calculated proliferation, fusion, and differentiation indices. Scale =50 µm. (f) Cytokine expression in cultured peritoneal macrophages from 3‐ and 20‐month‐old mice with and without LPS simulation (10 ng/mL, *n* = 3). (g) Schematic depiction of C2C12 culture conditions with young or old macrophage conditioned media. (H) C2C12 cells were treated with conditioned medium from young and old macrophages and proliferation was assessed (*n* = 3). (i–j) Differentiation and fusion index of C2C12 grown in 1:1 differentition:conditioned media from young and old macrophages (*n* = 3). (k) Representative images of BrdU^+^ (yellow) or Myh^+^ (yellow) cells used to calculated proliferation, fusion, and differentiation indices Scale =50 µm. All analyses were done using a one‐ or two‐way ANOVA. *p < 0.05, **p < 0.01, ***p < 0.001. Con, Control; LPS, Lipopolysaccharides; CM, Conditioned Media; SFM, Serum‐free media; DM, Differentiation media

We next assessed whether BM aging had an impact on host cell proliferation. We first established normal levels of satellite cell and mesenchymal stem cell (MSC) proliferation in WT mice. Satellite cells were more abundant in young mice, prior to injury (Figures 1J and 6C). Three days post‐CTX the number of BrdU^+^ satellite cells increased significantly in young but not old mice (Figure 5C). This coincided with an increase in MSC proliferation in young mice (Figure 5D). After BM transplant we did not detect any change in GFP^−^ satellite cell number (Figure S8) and no GFP^+^ satellite cells were detected in Y‐O or O‐O mice. Three days post‐CTX, GFP^−^ host cell proliferation in Y‐O and O‐O mice was assessed using BrdU and immunofluorescent staining. In response to injury, in both Y‐O and O‐O mice we detected BrdU^+^/GFP^−^ cells (stars); however, there were significantly more GFP^−^ proliferating cells in mice that received young BM (Figure 5E). This initial analysis suggested that immune rejuvenation improves host cell proliferation. To understand the source of this signal, we assessed satellite cell and MSC proliferation via flow cytometry. At 3 days post‐CTX the percentage of cells positive for satellite cell markers (α7‐integrin or VCAM‐1) was increased in Y‐O chimeras (Figure 5F and Figure S8). When normalized to muscle weight, the number of BrdU^+^ satellite cells was increased in Y‐O mice (Figure 5G). We also analyzed both the GFP^−^ and GFP^+^ MSC population three days after injury (Figure S9). There was a small fraction of BrdU^+^/GFP^+^ MSCs, which was higher in the Y‐O cohort, however this population was substantially lower than the number of host GFP^−^ MSCs (Figure S9A,B). In GFP^−^ MSCs, there was no change in the total cell number of BrdU^+^ cells, however the percentage of BrdU^+^ GFP^−^ MSCs after injury (Figure S9C). Together these data show that heterochronic BM chimerism increases host cell proliferation.

### 2.7 Aging alters the pro‐ and anti‐inflammatory expression profiles of activated macrophages to differentially regulate myoblast proliferation and differentiation

One of the proposed mechanisms of muscle repair is that pro‐inflammatory (M1) macrophages and cytokines promote myoblast proliferation (and inhibit differentiation) while anti‐inflammatory/immunomodulatory (M2) macrophages promote myogenesis (Arnold et al., 2007; Deng et al., 2012). Our BM chimerism and aging data showed that Ly6C^hi^ monocytes and pro‐inflammatory cytokines (e.g. *IL*‐*1β*) are part of the muscle repair process that deteriorate with age. To correlate cytokine expression to immune cell type, we analyzed microarray expression data from previously published data (Varga et al., 2016) of Gr1^+^ monocytes and Cx3cr1^hi^ macrophages isolated from CTX‐injured muscle of young mice at 1, 2, 4, and 8 days post‐injury (Figure S10). Both monocytes and macrophages expressed pro‐inflammatory cytokines such as *IL*‐*1β* and *TNFα* early in muscle repair, from 1 to 2 days post‐injury while *IL*‐*12b* and *IFNγ* are expressed at later stages from 4 to 8 days post‐injury. *IL*‐*10* expression patterns vary; in Gr1^+^ monocytes, *IL*‐*10* expression was higher at 1 and 2 days post‐injury while in Cx3cr1^hi^ macrophages showed biphasic expression at days 1, 2, and 8. Thus, cytokine expression within recruited monocyte and macrophage populations to the injured muscle exhibits temporal changes that correspond to our cytokine analyses using gross skeletal muscle tissue. Therefore, we next investigated how cytokines secreted by myeloid cells influence myogenesis. To do this we used an *in vitro* model using C2C12 myoblasts to study the effect myeloid‐derived signals have on myoblast proliferation and differentiation. To assess proliferation, cells were grown in serum‐free media containing BrdU and recombinant cytokines (IL‐12, IL‐10, IL‐1β, TNFα, or IFNγ) and the number of BrdU^+^ cells was counted. To assess differentiation and cell fusion, C2C12 myoblasts were cultured for 96 h in differentiation medium with the indicated cytokines and stained for the terminal differentiation marker, myosin heavy chain (Myh). Experimental design is depicted in the schematic in Figure 6A. IL‐12, IL‐1β, and TNFα increased myoblast proliferation. TNFα also had a potent role in suppressing fusion and differentiation (Figure 6B‐E). IL‐10 was the only cytokine tested that promoted the differentiation and fusion of myoblasts. IFNγ had no significant effect on C2C12 proliferation or differentiation.

To determine age‐related defects of expression of these cytokines, we cultured peritoneal macrophages from young or old mice in vitro and evaluated cytokine expression after activation with lipopolysaccharides (LPS). Expression of *IL*‐*1β*, *IL*‐*12b*, and *IL*‐*23* was consistently higher in young activated macrophages compared with old activated macrophages (Figure 6F). *TNFα*, *IFNγ*, and *IL*‐*10* expression showed the opposite trend and were more highly expressed in old LPS‐activated macrophages. To determine how macrophage age may affect myoblast function, C2C12 myoblasts were cultured in conditioned medium (CM) collected from young or old activated macrophages (Figure 6G). Compared to serum‐free control, CM from young macrophages had no effect on myoblast proliferation but CM from old LPS‐stimulated macrophages reduced cell proliferation (Figure 6H,K). We next cultured C2C12 cells under differentiation conditions using 1:1 differentiation and conditioned medium for 96 h (Figure 6G). Under these long‐term culture conditions, myogenesis proceeded at a slower rate than with 100% differentiation medium (not shown) or control conditions (Figure 6I‐K). Notably, CM from young macrophages had a stronger effect on repressing myogenesis than CM from old macrophages. These in vitro studies demonstrate a mechanism whereby aging initiates a different cytokine response following activation, which in turn impacts the timing of myogenic repair processes such as myoblast proliferation and differentiation.

## 3 DISCUSSION

The appreciation of inflammatory pathways as beneficial to tissue repair is growing, and harnessing the activity of macrophages, monocytes, and T cells is being recognized as potential treatment strategies for numerous conditions. In skeletal muscle, the role of the inflammatory response in tissue repair has been investigated using various means of muscle damage in young mice; however, the impact that aging has on this process is unclear. Recent studies have begun to dissect the impact that aging has on muscle repair by investigating intrinsic and extrinsic properties of satellite cells, the mediators of myogenesis. Here, we show that CTX induces a rapid and trainset inflammatory response that is impaired with age and is associated with diminished myogenic capacity. Replacement of old BM with young BM restored the timing of the inflammatory cascade to a more youthful profile, and this increased satellite cell proliferation in old mice. In vitro, myoblast proliferation was reduced when cultured in CM from old macrophages, while CM from young macrophages significantly impaired C2C12 differentiation. Together, these data highlight that aging of the immune system controls the decision between proliferation and myogenesis and changes in these factors can hinder different phases of muscle repair.

Shavlakadze et al. profiled gene expression in the gastrocnemius, liver, kidney, and hippocampus across the lifespan of the rat from 6 to 27 months old and found that inflammation is a universal part of tissue aging (Shavlakadze et al., 2019). Our flow cytometry and gene expression profiles concur that old muscles are enriched in inflammatory immune cells and cytokines. In uninjured muscle, we observed more neutrophils and T cells and fewer satellite cells in old mice as well as greater expression of *IL*‐*1β*, *IFNγ*, and *IL*‐*12b*. In contrast, after injury our gene and cell profiles demonstrate depressed/delayed expression of pro‐inflammatory cytokines, fewer leukocytes (specifically myeloid cells) and reduced satellite cell and MSC proliferation in old muscle. This is in general agreement with a previous study that observed delayed inflammation and myogenesis in mice that received heterochronic autografts from young or geriatric mice (Shavlakadze et al., 2010). We did not, however, observe delayed myogenesis in old mice but a general impairment in early and late myogenic gene expression.

The function of old satellite cells has been shown by others to be dependent upon its environment: studies from the late 80’s used muscle grafts to show that old muscle performs better in a young environment while in contrast, young muscle grafts perform poorly in old hosts (Carlson, 1989) and more recently parabiosis and blood transfusion has shown that young blood can improve skeletal muscle repair as assessed by improved regeneration or satellite cell proliferation 5 days after CTX (Conboy et al., 2005; Rebo et al., 2016). As detailed in young and old mice, the inflammatory and myogenic responses to injury are significantly altered with aging. As T cells, neutrophils, monocytes, and macrophages are necessary for muscle repair and are important regulators of myogenesis (Arnold et al., 2007; Saclier et al., 2013), we expected that transplanting young BM into old mice would improve both the immune cell response and endogenous satellite cell proliferation. Wang et al. recently performed heterochronic BM transplant and observed increased muscle mass by young BM cells in old mice while old BM had a negative impact on satellite cell function in young mice (Wang et al., 2019). Their findings were analyzed based on uninjured muscle tissue. In our study, Y‐O chimeras showed an overall improvement in gross muscle mass and behavioral and locomotive tests; however, we did not see any change in satellite cell number or inflammation at baseline. Three days after CTX, mice that received young BM demonstrated patterns of a young immune response, and while we observed improvements in satellite cell proliferation, the magnitude of change was small and significantly less than the rates of satellite cell proliferation seen in young mice. We also observed higher expression of *MyoG* and *Myh3* in Y‐O mice; however, these changes were modest, and we could not detect any regenerated fibers at 10 days post‐CTX either by staining for Myh3 or quantification of centrally nucleated myofibers (data not shown). Thus, while others who have shown successful myogenic rejuvenation strategies in old muscle, our BM chimera model may not be as conducive to rejuvenation. One potential explanation for this is the experimental model itself, which utilizes lethal irradiation to empty the BM compartment. This procedure could simultaneously damage satellite cell genomic integrity, precluding environmental rejuvenation. Alternatively, unlike whole muscle graft, parabiosis, or blood transfusion, we studied specifically how young BM cells may rejuvenate myogenesis in old muscle. It is possible that the factors and/or cells targeted in other models of rejuvenation are more robust at changing the microenvironment.

Global impairment of monocyte and macrophage recruitment using liposomal clodronate, CCR2^(−/−)^ knockout mice or CCR2^(−/−)^ BM transplant leads to significant deficits in muscle repair (Martinez et al., 2010; Summan et al., 2006; Sun et al., 2009) indicating inflammation and resolution are important to tissue healing. More detailed investigation of the early and late phases of muscle repair is required to better optimize the transition between proliferation and differentiation. For example, an important feature of muscle repair that has been well‐documented is that differential activation of macrophages to pro‐ or anti‐inflammatory/immunomodulatory states (e.g., polarization to classical M1 or M2 macrophage phenotypes) favor myoblast proliferation or myogenesis, respectively (Arnold et al., 2007; Saclier et al., 2013). IL‐10 is a classical M2 marker which can limit macrophage proliferation (O’Farrell et al., 2000) and inflammatory cytokine production (Wang et al., 1995). At 3 days post‐injury, *IL*‐*10* was upregulated in old but not young muscle and this trend was associated with significant accumulation of M2 macrophages to injured muscle. In our Y‐O chimeras, M2 macrophage accumulation was reduced compared with O‐O mice after injury, reflecting a rejuvenated phenotype. In vitro, LPS‐stimulated macrophages from old mice favored M2 polarization characteristics and expressed lower levels of pro‐inflammatory cytokines (e.g., *IL*‐*1β*) but higher levels of *IL*‐*10*. One may expect that upregulation of an anti‐inflammatory cytokine to be beneficial to overall tissue recovery. Indeed, others have shown that IL‐10 is required for myogenesis post‐injury (Deng et al., 2012) and muscle‐specific expression of IL‐10 in old muscle reduces inflammation and insulin resistance (Dagdeviren et al., 2017). However, IL‐10 can also suppress the M1 response by downregulation of M1 cytokines including IFNγ, IL‐1β, and IL‐12b (Gazzinelli et al., 1996; Kobayashi et al., 2012; Watford et al., 2004) which as we have shown in vitro could alter myoblast proliferation. With respect to aging, *IL*‐*10* expression is upregulated in old muscle and associated with fibrosis via overactivation of M2a macrophages (Wang et al., 2015). Additionally, M2 macrophages increase while M1 macrophages decrease with aging in human muscles (Cui et al., 2019), demonstrating the upregulation of the M2 macrophage response in aging is detrimental to muscle health and/or repair and that restoration of the M1 response may be key to maintenance of muscle repair with age.

Targeting age‐dependent changes in macrophage populations (or other immune cells) could impact cytokines that regulate myoblast proliferation, such as IL‐1β, and alter the timing between proliferation and differentiation in satellite cells; however, this intercellular communication is not limited to inflammatory molecules. For example, the macrophage‐derived secreted protein, Adamts1, promotes satellite cell proliferation via inhibition of Notch signaling to release satellite cells from quiescence (Du et al., 2017). It was also recently shown that macrophages can sense and respond to metabolites produced in muscle. Shang et al. demonstrated that age‐ and injury‐related decreases in glutamine are compensated for by macrophages, which produce glutamine that is taken up by satellite cells, facilitating their proliferation and differentiation (Shang et al., 2020).

## 4 CONCLUSION

Together our data indicate that muscle repair processes in young mice invoke a pro‐inflammatory microenvironment conducive to satellite cell and MSC proliferation. Aging leads to chronic muscle inflammation in resting muscle, yet in response to acute injury the inflammatory response is dampened. Instead, anti‐inflammatory/immunomodulatory (i.e., M2) macrophages accumulate, which may limit satellite cell proliferation in part through IL‐10. Heterochronic bone marrow chimerism restored the timely infiltration of myeloid cells to injured muscle and improved satellite cell proliferation. These data identify the temporal response of immune cell and muscle‐resident cell activation and demonstrate the importance of BM aging (extrinsic factors) on the microenvironment of old skeletal muscle in satellite cell activation and differentiation.

## 5 EXPERIMENTAL PROCEDURES

Experimental procedures can be found in supporting information online methods.

## CONFLICT OF INTEREST

None.

## AUTHOR CONTRIBUTIONS

S.W.T. and F.A designed the experiments. S.W.T wrote the manuscript. S.W.T, F.A., L.W., A.Y., and J.W performed the bone marrow reconstitution experiments. S.W.T and A.Y. performed immunofluorescence experiments. L.W. and S.M. performed and analyzed open field and rotarod experiments. S.W.T. and F.A. performed flow cytometry experiments. S.W.T performed gene expression analysis, C2C12 myoblast experiments and CTX treatments. F.A. performed *in vitro* experiments involving peritoneal macrophages. F.A. and R‐K. L. edited the manuscript.

## Supporting information

Supplementary Material

Supplementary Material

## Data Availability

The data that support the findings of this study are available in at Gene Expression Omnibus at https://www.ncbi.nlm.nih.gov/geo/, reference number GSE71152.
