# Supplementary material for: Delineating the relationship between immune system aging and myogenesis in muscle repair
Source: Aging Cell. 2021 Jan 28;20(2):e13312. doi: 10.1111/acel.13312 (PMC7884032; doi:10.1111/acel.13312)
Supplement: Supplementary file 1 — Supplementary Material [file ACEL-20-e13312-s001.pdf]

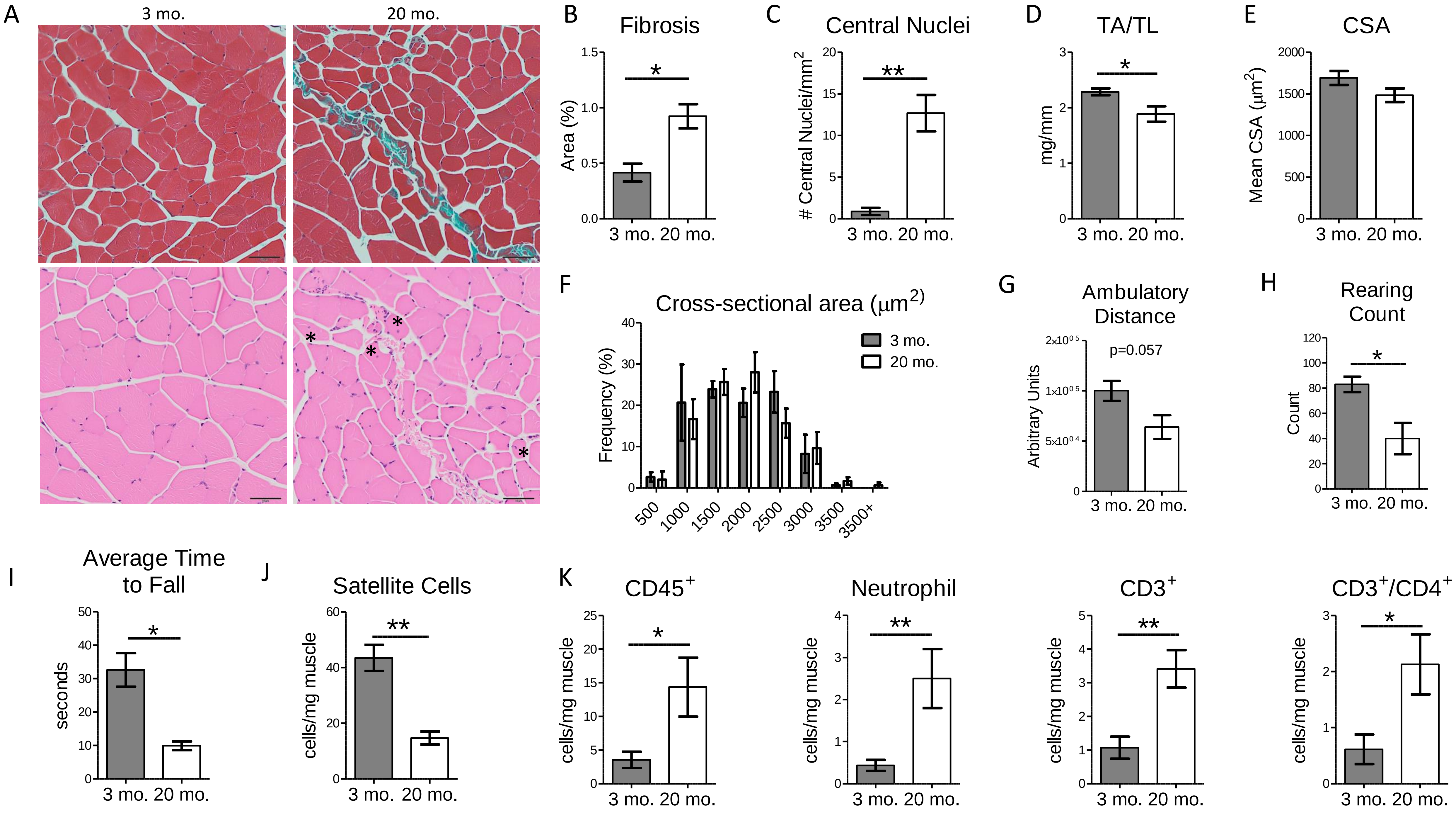
A
3 mo.
20 mo.
B
Fibrosis
Area (%)
3 mo. 20 mo.
C
Central Nuclei
# Central Nuclei/mm2
D
TA/TL
mg/mm
E
CSA
Mean CSA (μm2)
F
Cross-sectional area (μm2)
Frequency (%)
3 mo.
20 mo.
500 1000 1500 2000 2500 3000 3500 3500+
G
Ambulatory Distance
p=0.057
Arbitrary Units
H
Rearing Count
Count
I
Average Time to Fall
seconds
J
Satellite Cells
cells/mg muscle
K
CD45+
Neutrophil
CD3+
CD3+/CD4+
cells/mg muscle

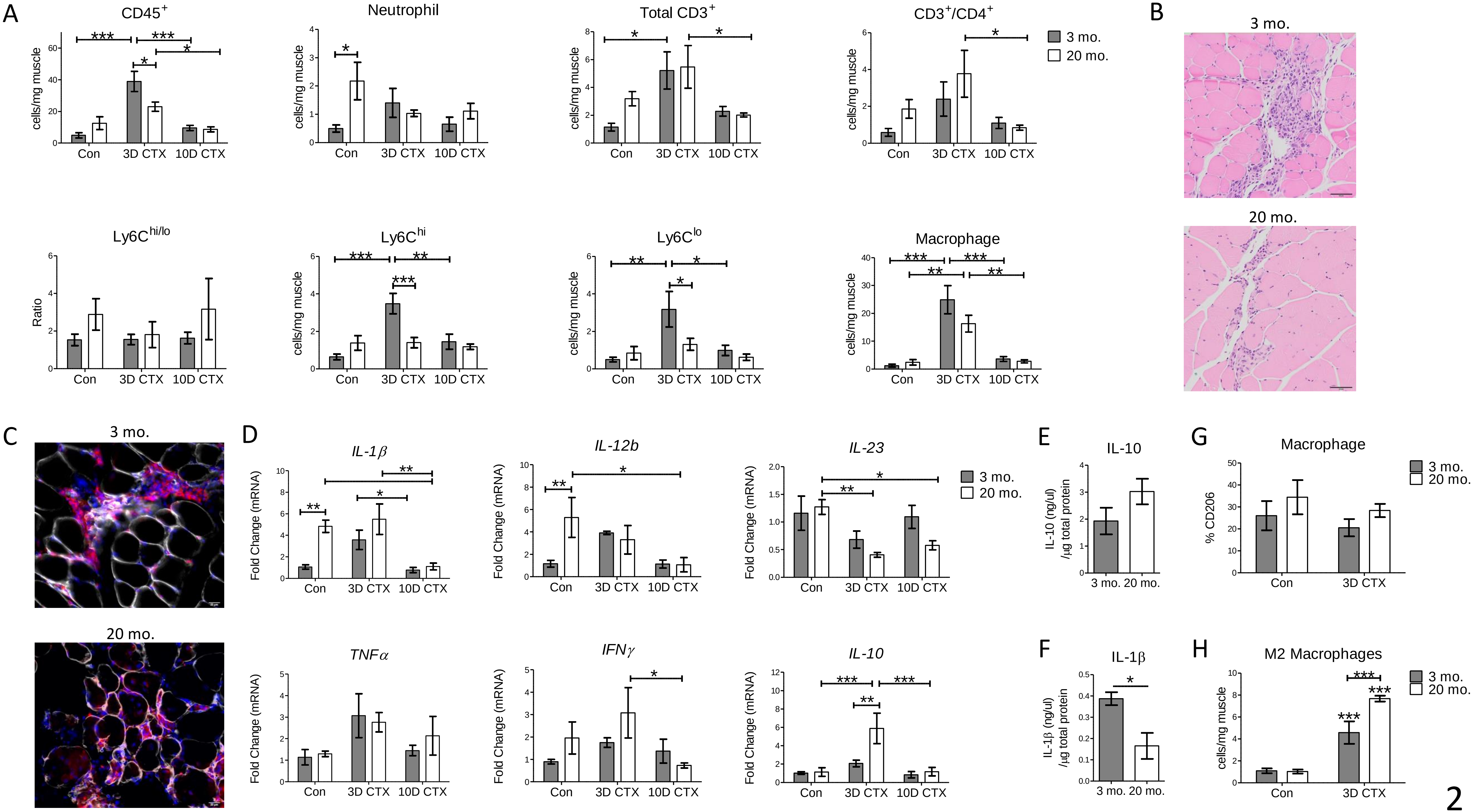
A
CD45+
Neutrophil
Total CD3+
CD3+/CD4+
3 mo.
20 mo.
cells/mg muscle
Con
3D CTX
10D CTX
Ly6Chi/lo
Ratio
Ly6Chi
Ly6Clo
Macrophage
B
3 mo.
20 mo.
C
3 mo.
20 mo.
D
IL-1β
IL-12b
IL-23
TNFα
IFNγ
IL-10
Fold Change (mRNA)
E
IL-10
IL-10 (ng/ul) /μg total protein
F
IL-1β
IL-1β (ng/ul) /μg total protein
G
Macrophage
% CD206
H
M2 Macrophages

2

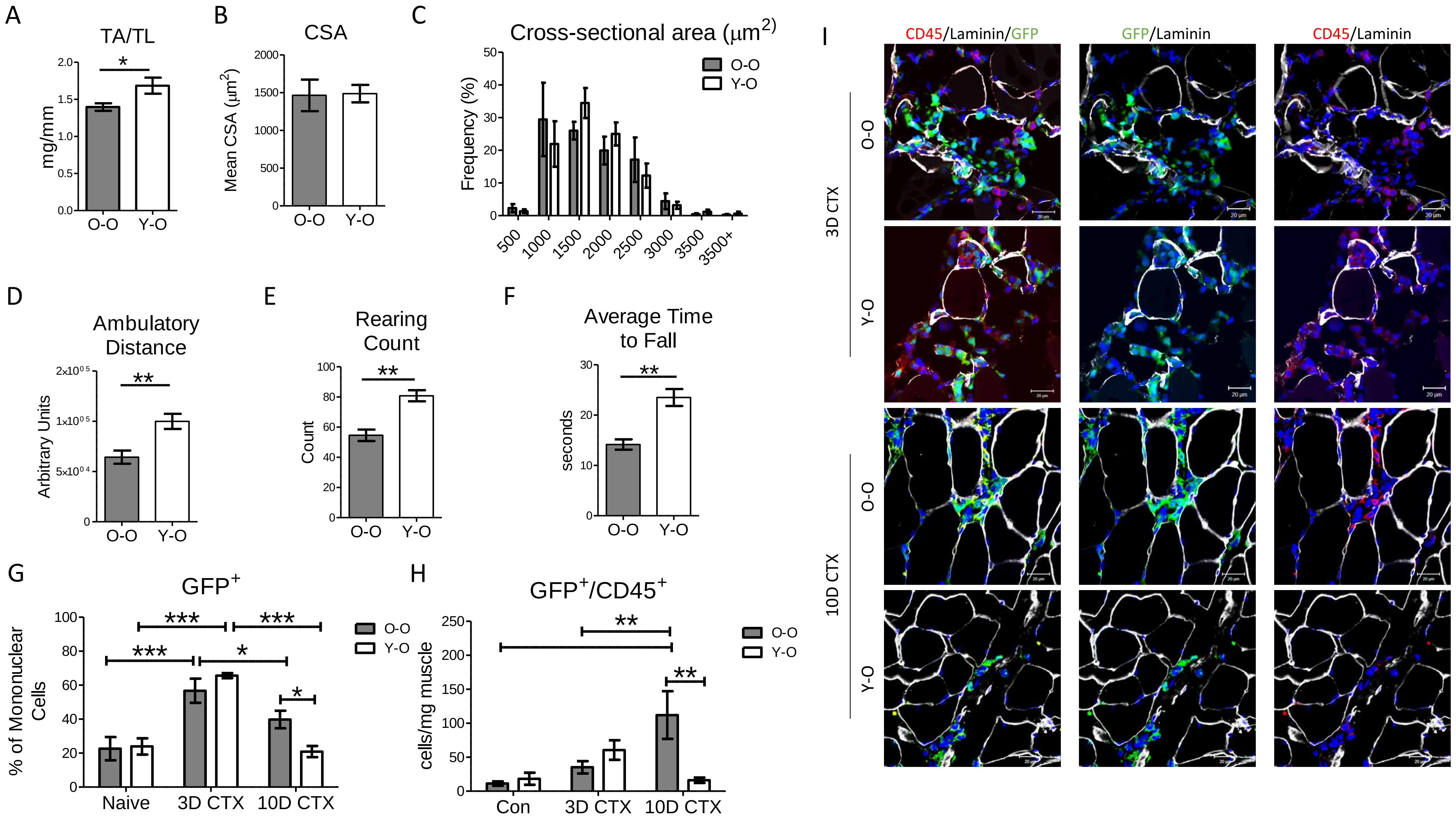
A
TA/TL
mg/mm
O-O
Y-O
B
CSA
Mean CSA (μm2)
C
Cross-sectional area (μm2)
Frequency (%)
O-O
Y-O
500
1000
1500
2000
2500
3000
3500
3500+
D
Ambulatory Distance
Arbitrary Units
E
Rearing Count
Count
F
Average Time to Fall
seconds
G
GFP+
% of Mononuclear Cells
Naive
3D CTX
10D CTX
H
GFP+/CD45+
cells/mg muscle
Con
I
CD45/Laminin/GFP
GFP/Laminin
CD45/Laminin
3D CTX
10D CTX

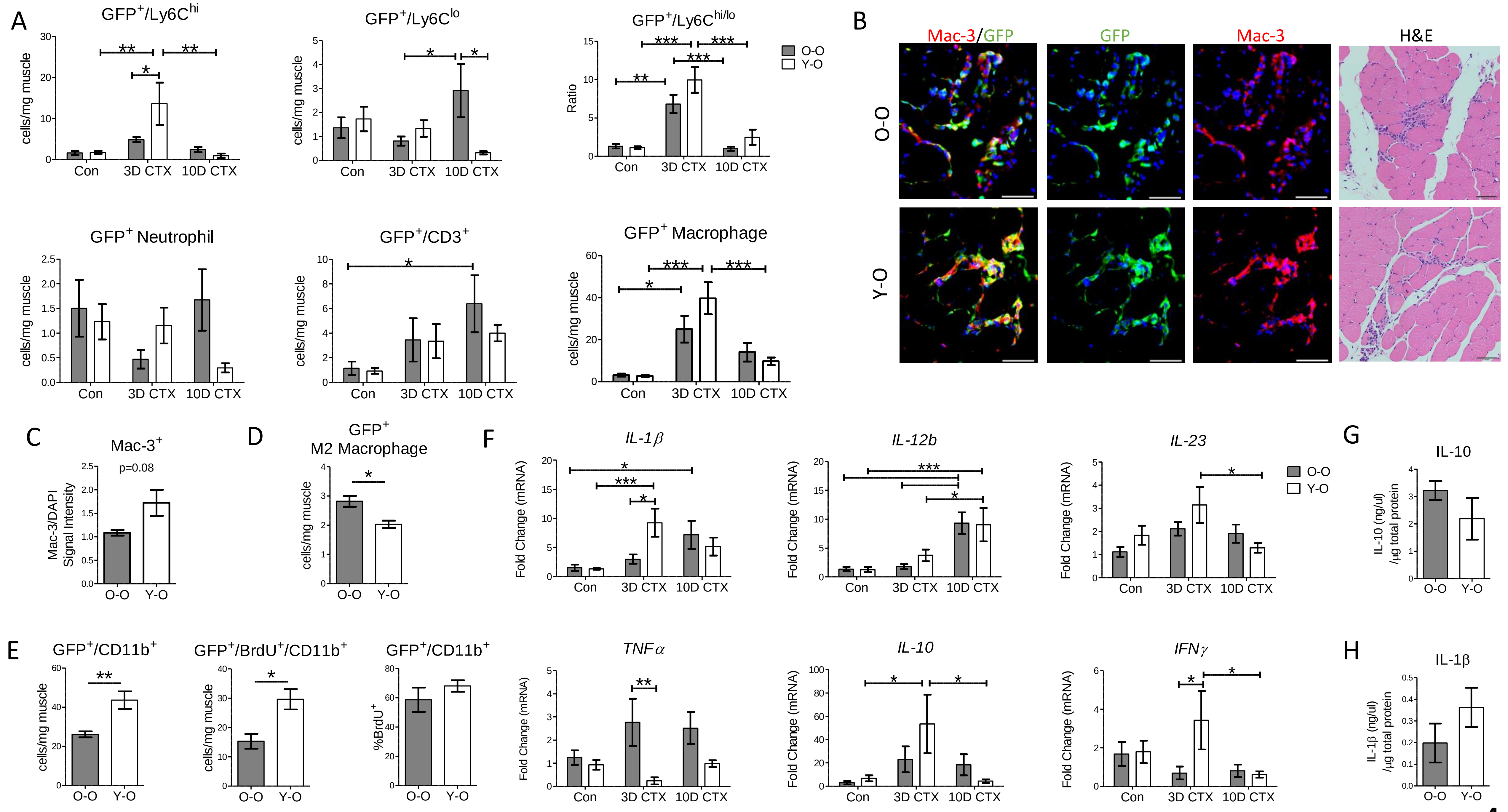
A
GFP+/Ly6Chi
cells/mg muscle
Con
3D CTX
10D CTX
GFP+/Ly6Clo
GFP+/Ly6Chi/lo
Ratio
O-O
Y-O
GFP+ Neutrophil
GFP+/CD3+
GFP+ Macrophage
B
Mac-3/GFP
GFP
Mac-3
H&E
O-O
Y-O
C
Mac-3+
p=0.08
Mac-3/DAPI Signal Intensity
D
GFP+ M2 Macrophage
F
IL-1β
IL-12b
IL-23
Fold Change (mRNA)
G
IL-10
IL-10 (ng/ul) /µg total protein
E
GFP+/CD11b+
GFP+/BrdU+/CD11b+
%BrdU+
TNFα
IL-10
IFNγ
H
IL-1β
IL-1β (ng/ul) /µg total protein

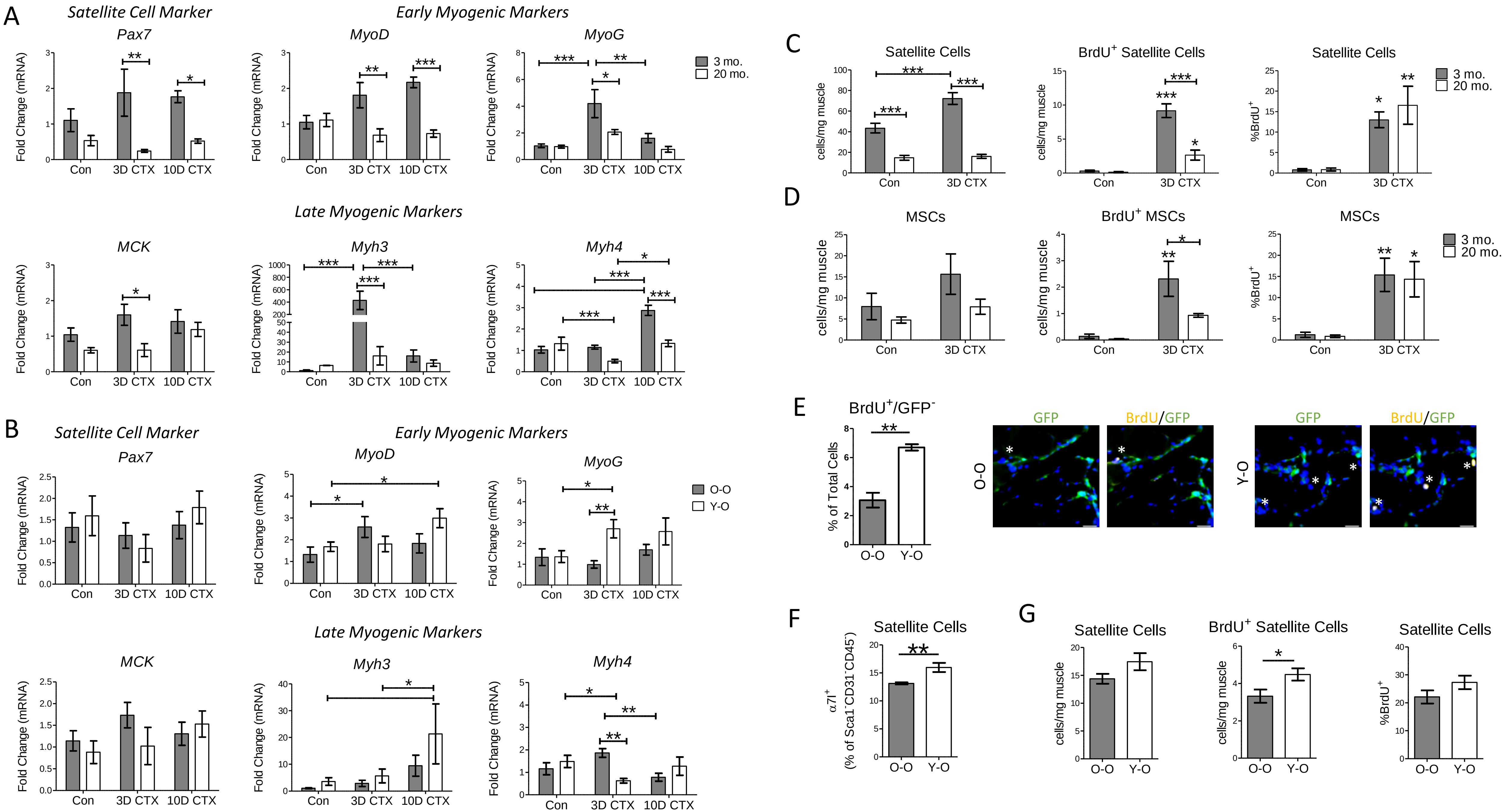
A
Satellite Cell Marker
Pax7
Early Myogenic Markers
MyoD
MyoG
Late Myogenic Markers
MCK
Myh3
Myh4
Fold Change (mRNA)
Con
3D CTX
10D CTX
3 mo.
20 mo.
B
Satellite Cell Marker
Pax7
Early Myogenic Markers
MyoD
MyoG
Late Myogenic Markers
MCK
Myh3
Myh4
O-O
Y-O
C
Satellite Cells
BrdU+ Satellite Cells
cells/mg muscle
%BrdU+
D
MSCs
BrdU+ MSCs
E
BrdU+/GFP-
% of Total Cells
GFP
BrdU/GFP
F
α7I+ (% of Sca1-CD31-CD45-)
G
Satellite Cells
BrdU+ Satellite Cells

5

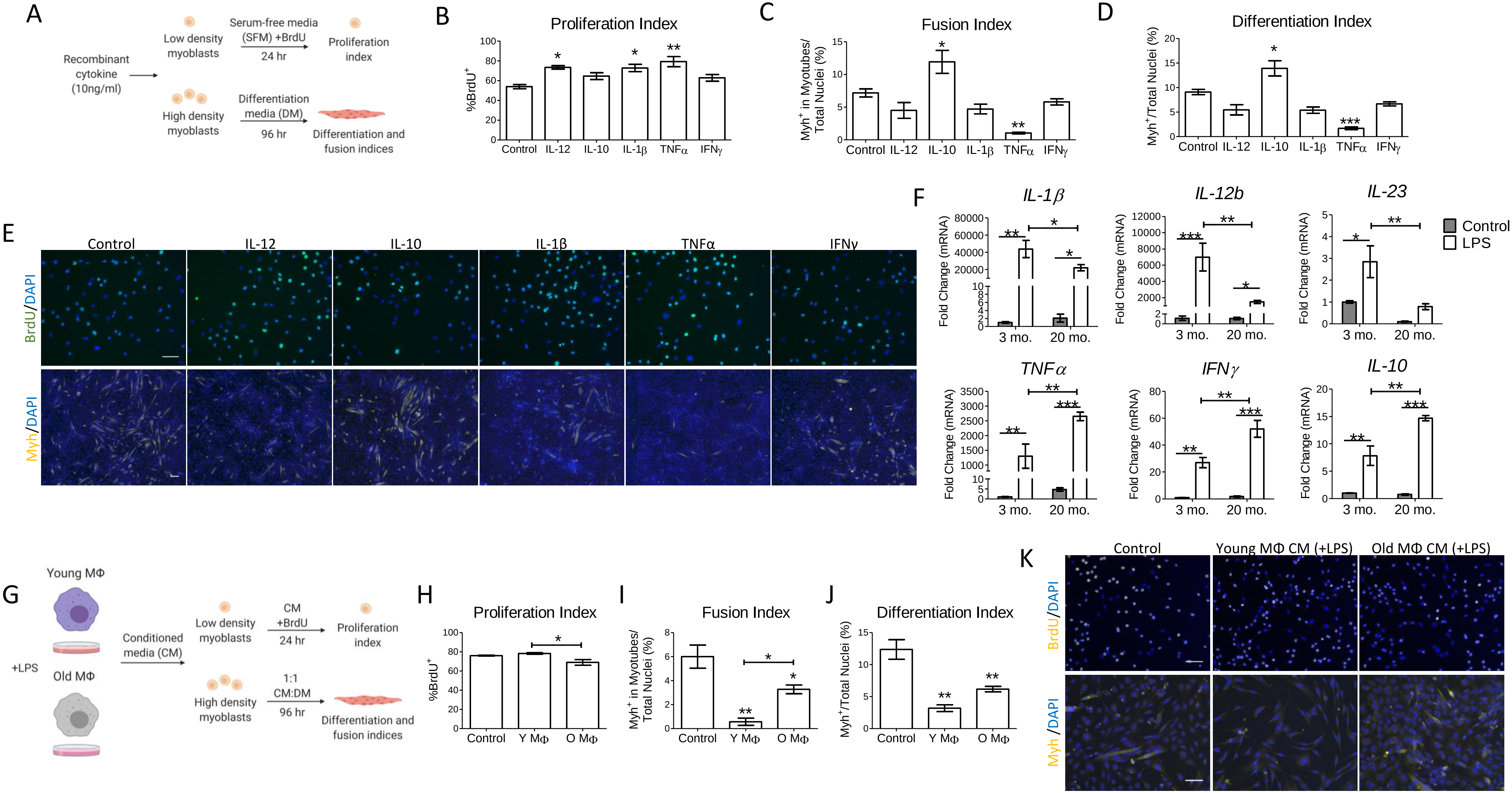
A
Recombinant cytokine (10ng/ml)
Low density myoblasts
Serum-free media (SFM) +BrdU
24 hr
Proliferation index
High density myoblasts
Differentiation media (DM)
96 hr
Differentiation and fusion indices
B
Proliferation Index
%BrdU+
Control
IL-12
IL-10
IL-1β
TNFα
IFNγ
C
Fusion Index
Myh+ in Myotubes/ Total Nuclei (%)
D
Differentiation Index
Myh+/Total Nuclei (%)
E
BrdU/DAPI
Myh/DAPI
F
IL-1β
IL-12b
IL-23
TNFα
IFNγ
IL-10
Fold Change (mRNA)
3 mo.
20 mo.
Control
LPS
G
Young MΦ
Old MΦ
+LPS
Conditioned media (CM)
Low density myoblasts
CM +BrdU
24 hr
Proliferation index
High density myoblasts
1:1 CM:DM
96 hr
Differentiation and fusion indices
H
Proliferation Index
%BrdU+
Control
Y MΦ
O MΦ
I
Fusion Index
Myh+ in Myotubes/ Total Nuclei (%)
J
Differentiation Index
Myh+/Total Nuclei (%)
K
Control
Young MΦ CM (+LPS)
Old MΦ CM (+LPS)
BrdU/DAPI
Myh /DAPI

# Supplemental Information

## Supplemental Table 1. Primers used for qRT-PCR (mouse).

| | Forward | Reverse |
|---|---|---|
| β-actin | TGTTACCAACTGGGACGACA | AAGGAAGGCTGGAAAAGAGC |
| Hprt | CGGCACAGTCAAGGCCGAGAATGG | TCATGGATGACCTTGGCCAGGGGG |
| IFNγ | AGCAAGGCGAAAAAGGATGC | TCATTGAATGCTTGGCGCTG |
| IL-10 | CCAAGCCTTATCGGAAATGA | TTTTCACAGGGGAGAAATCG |
| IL-12b (IL-12p40) | TGGTTTGCCATCGTTTTGCTG | ACAGGTGAGGTTCACTGTTTCT |
| IL-1β | CAGGCAGGCAGTATCACTCA | AGGCCACAGGTATTTGTCG |
| IL-23 | TCCCTACTAGGACTCAGCCAACTC | ACTCAGGCTGGGCACTG |
| MCK | AGTCCTACACGGTCTTCAAGG | AGGAAGTGGTCATCAATGAGC |
| Myh3 | CTTCACCTCTAGCCGGATGGT | AATTGTCAGGAGCCACGAAAAT |
| Myh4 | ACAGACTAAAGTGAAAGCC | CTCTCAACAGAAAGATGGAT |
| MyoD | TGGGATATGGAGCTTCTATCGC | GGTGAGTCGAAACACGGGTCAT |
| MyoG | CAGCTCCCTCAACCAGGAG | GACTGCAGGAGGCGCTGT |
| Pax7 | CTGGATGAGGGCTCAGATGT | GGTTAGCTCCTGCCTGCTTA |
| TNFα | GTAGCCCACGTCGTAGCAAAC | GCACCACTAGTTGGTTGTCTTTGA |

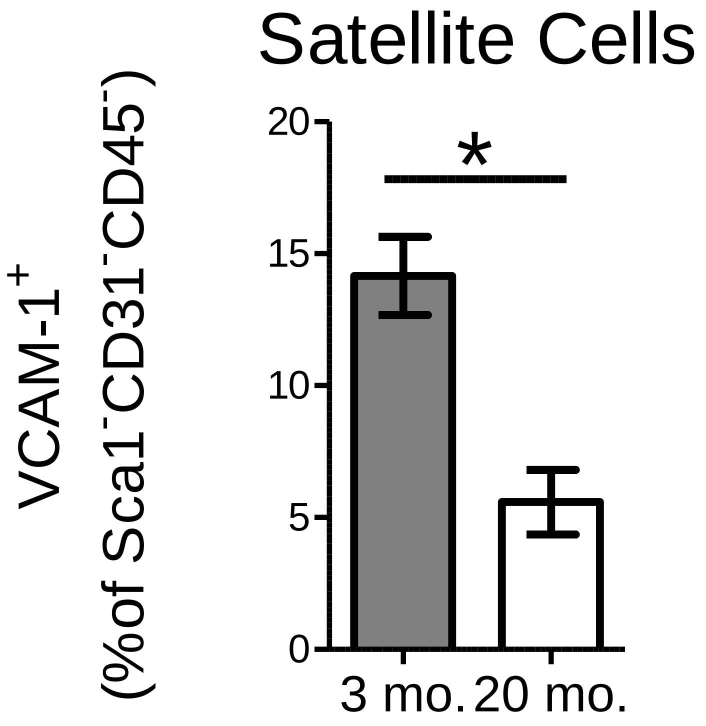


Supplemental Figure S1. Flow cytometry analysis of satellite cells from uninjured hindlimb muscle of young (3 month) and old (20 month) old mice (n=3). All data are presented as mean±SEM. Analysis was done using an unpaired t-test. *P<0.05

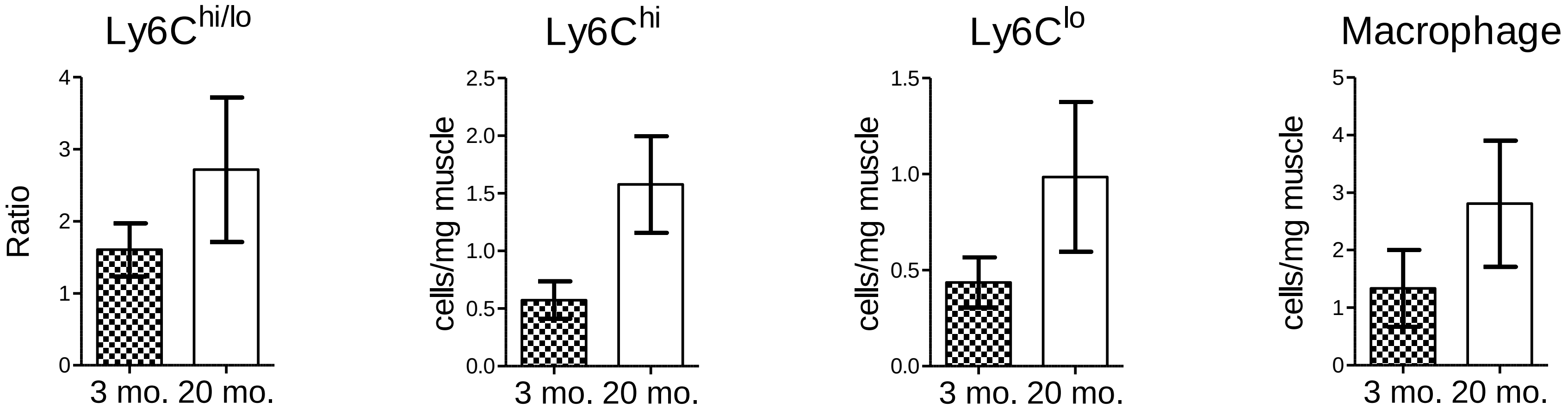


Supplemental Figure S2. Identification of immune cells by flow cytometry from uninjured hindlimb muscle of young (3 month) and old (20 month) old mice (n=5). All cells are plotted as cells/mg muscle. All data are presented as mean±SEM. All analyses were done using an unpaired t-test. No statistical difference was observed.

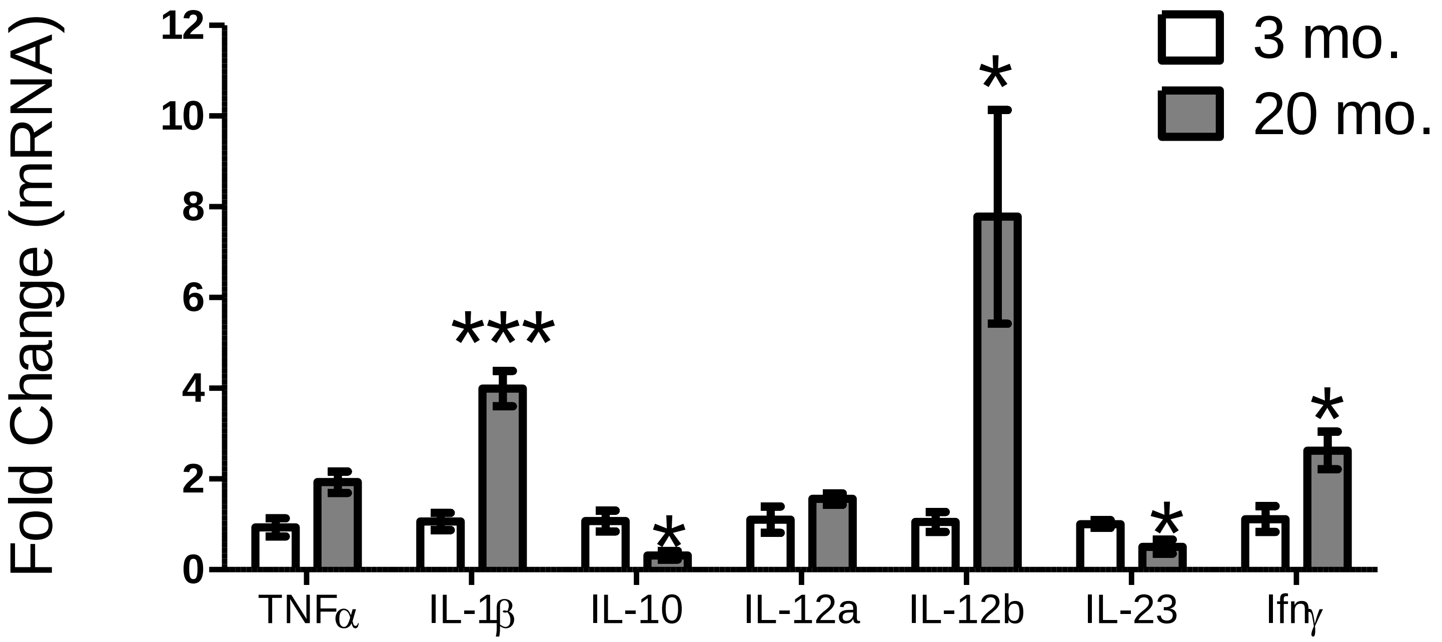


Supplemental Figure S3. Cytokine expression in young (3 month) and old (20 month) gastrocnemius muscles (n=4). The data are reported as relative to housekeeping gene, β-actin. All data are presented as mean±SEM. All analyses were done using an unpaired t-test. *$P<0.05$, **$P<0.01$, ***$P<0.001$.

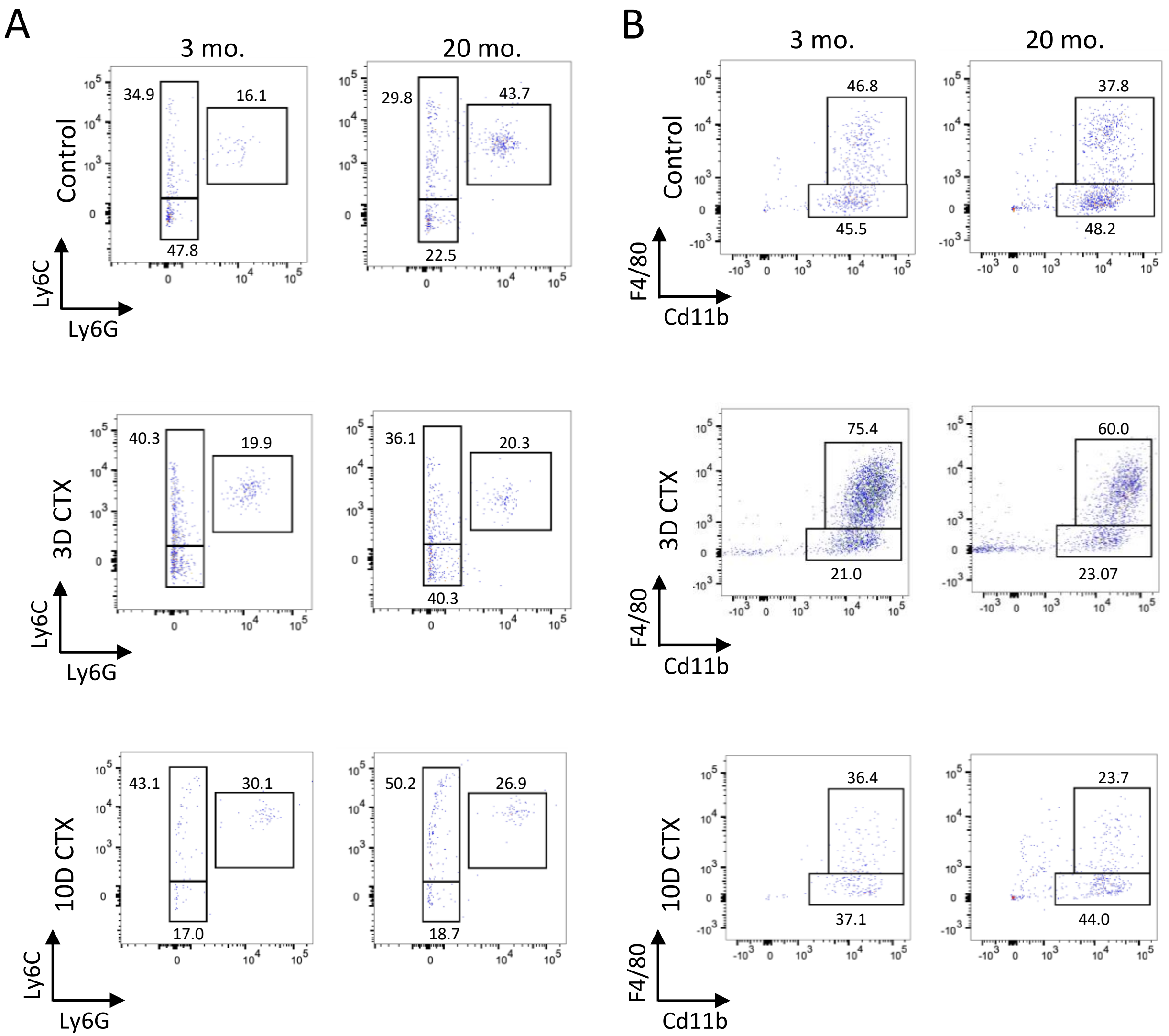


Supplemental Figure S4. Representative flow cytometry gating of macrophages (A), Ly6C$^{hi}$ and Ly6C$^{lo}$ monocytes and neutrophils (B) in young (3 month) and old (20 month) mice in response to CTX (cardiotoxin) injury at 3- and 10-days post CTX injury. Control = uninjured muscle. Percentages of different cell populations are shown by the indicated box.

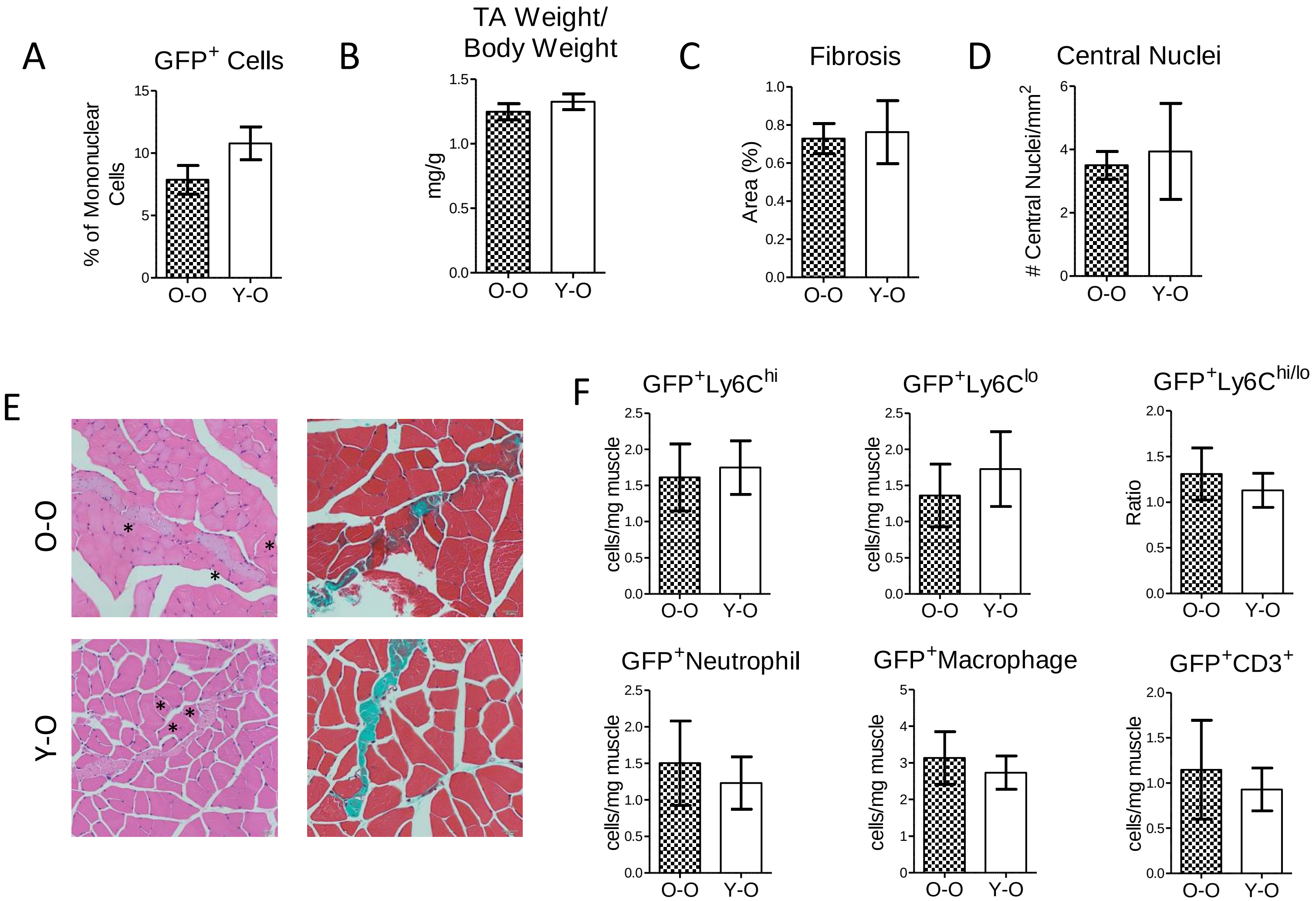


Supplemental Figure S5. Physiological and cellular changes in hindlimb muscle after heterochronic bone marrow transplant. A) The number of total GFP⁺ cells normalized to all mononuclear cells (n=8) in hind limb muscle as determined by flow cytometry. B) TA weight (normalized to body length (n=9). C) Quantification of intramuscular fibrosis (n=3). D) Quantification of muscle degeneration/regeneration via the number of central nuclei (n=3). E) Representative H&E or Trichrome images from O-O or Y-O uninjured muscle tissue. Scale = 20 µm. *Indicates centrally nucleated myofibers. F) The number of GFP⁺ immune cells (indicated by title) normalized to total muscle weight (milligram of muscle, n=4) as determined by flow cytometry. All data are presented as mean±SEM. All analyses were done using an unpaired t-test. No statistical differences were observed.

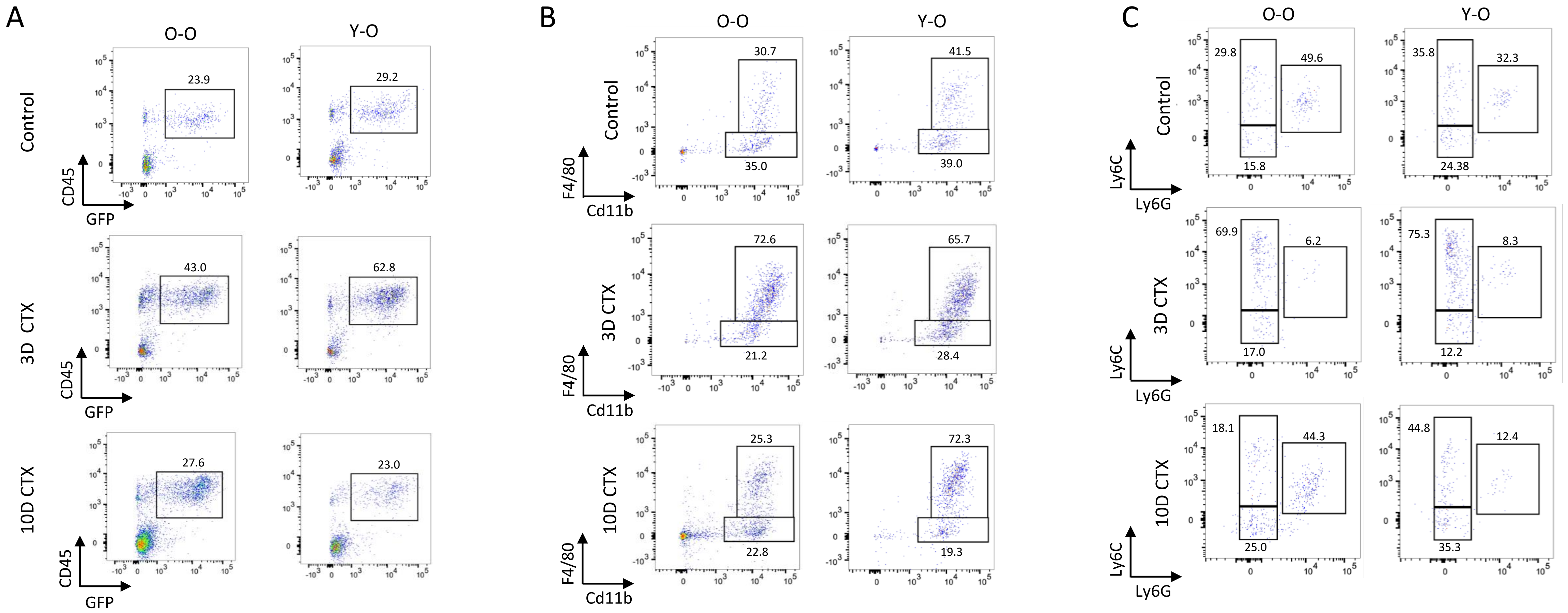


Supplemental Figure S6. Representative gating of immune cells in Y-O or O-O chimeras in response to CTX (cardiotoxin) injury. S6A depicts CD45 and GFP gating. S6B depicts macrophage gating. S6C depicts monocyte and neutrophil gating. Control = uninjured muscle. Percentages of different cell populations are shown by the indicated box.

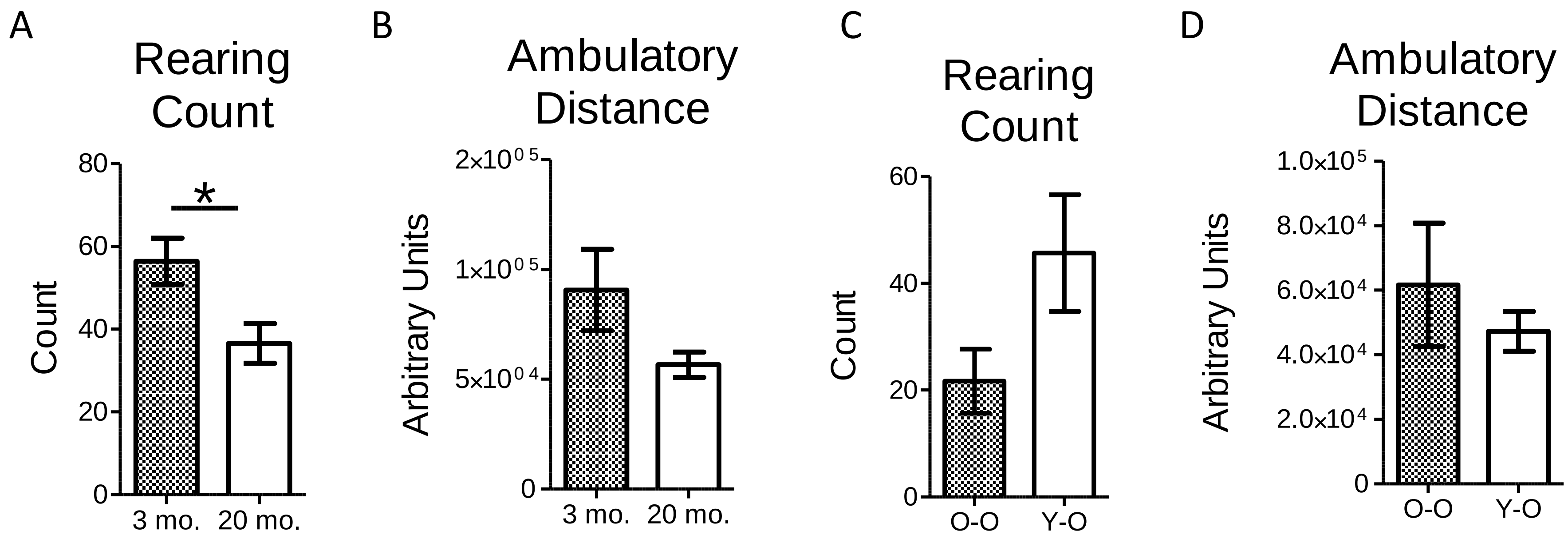


Supplemental Figure S7. Functional measurements of muscle strength as assessed by rearing count and ambulatory distance 7 days after CTX injury in WT mice (S7A-B) or chimeras (S7C-D). S6A-B) WT young (3 month) and old (20 month) old mice (n=5). S6C-D) All recipients (hosts) were >18 months old at the time of reconstitution and underwent a 12-week recovery period. Mice that received young $GFP^+$ $Sca\text{-}1^+$ BM cells are labelled Y-O. Mice that received old $GFP^+$ $Sca\text{-}1^+$ BM cells are labelled O-O (n=3). All data are presented as mean±SEM. All analyses were done using an unpaired t-test. *P<0.05.

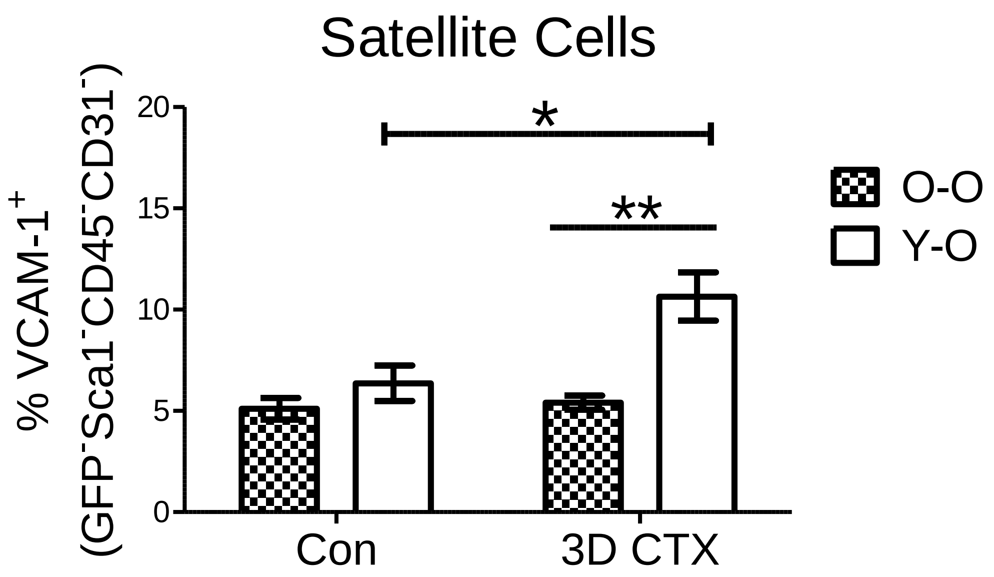


Supplemental Figure S8. Flow cytometry analysis of GFP$^-$ satellite cells from Y-O or O-O mice at 3D post CTX (n=4). Con = uninjured muscle tissue. All data are presented as mean±SEM. Data were analyzed using a 2-way ANOVA. *P<0.05 **P<0.01.

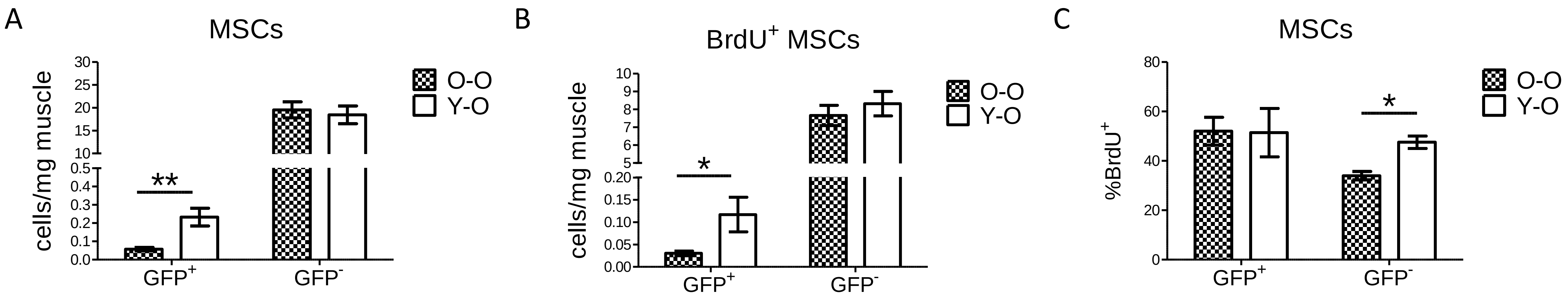


Supplemental Figure S9. Flow cytometry analysis of proliferating GFP$^-$MSCs and GFP$^+$MSCs from Y-O or O-O mice at 3D post CTX (n=5). Data are shown as total MSCs (A), BrdU$^+$ MSCs (B) or percent positive BrdU$^+$ MSCs (C). All data are presented as mean±SEM. All analyses were done using an unpaired t-test. *P<0.05. No statistical comparison between GFP$^+$MSCs and GFP$^-$MSCs was completed however they were plotted on the same graph to show the magnitude of difference in these populations of MSCs.

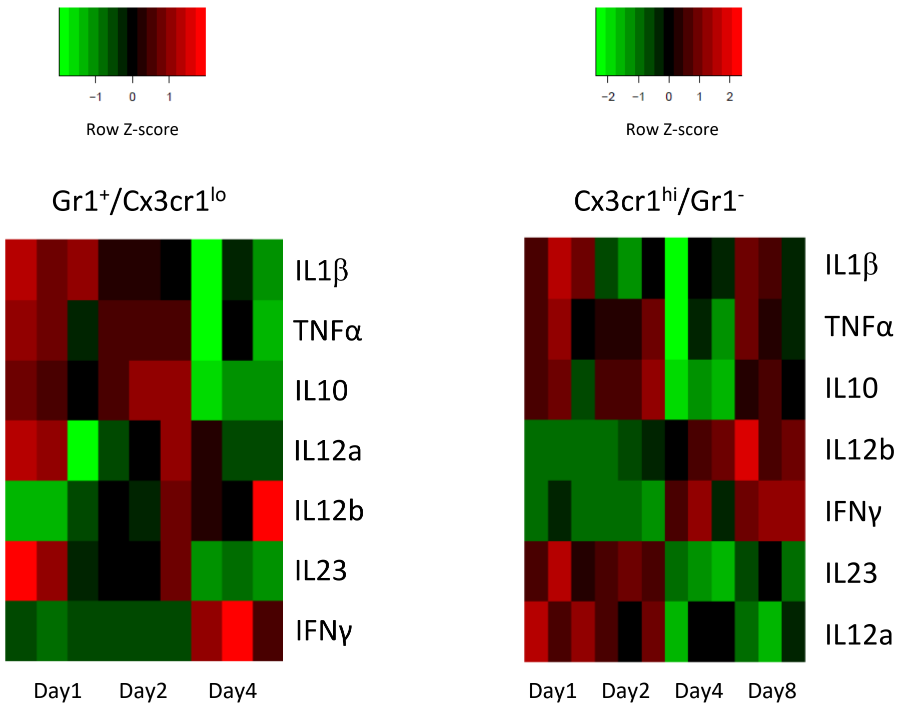


Supplemental Figure S10. Heatmap depictions of microarray gene expression values from Gr1$^{+}$/Cx3cr1$^{lo}$ or Cx3cr1$^{hi}$/Gr1$^{-}$ cells isolated from CTX injured muscle (Varga et al. 2016; GSE71152). Heatmaps were generated in RStudio. The timepoint of each analysis is indicated at the bottom of each heatmap (n=3). Day post injury: 1, 2, 4 or 8.

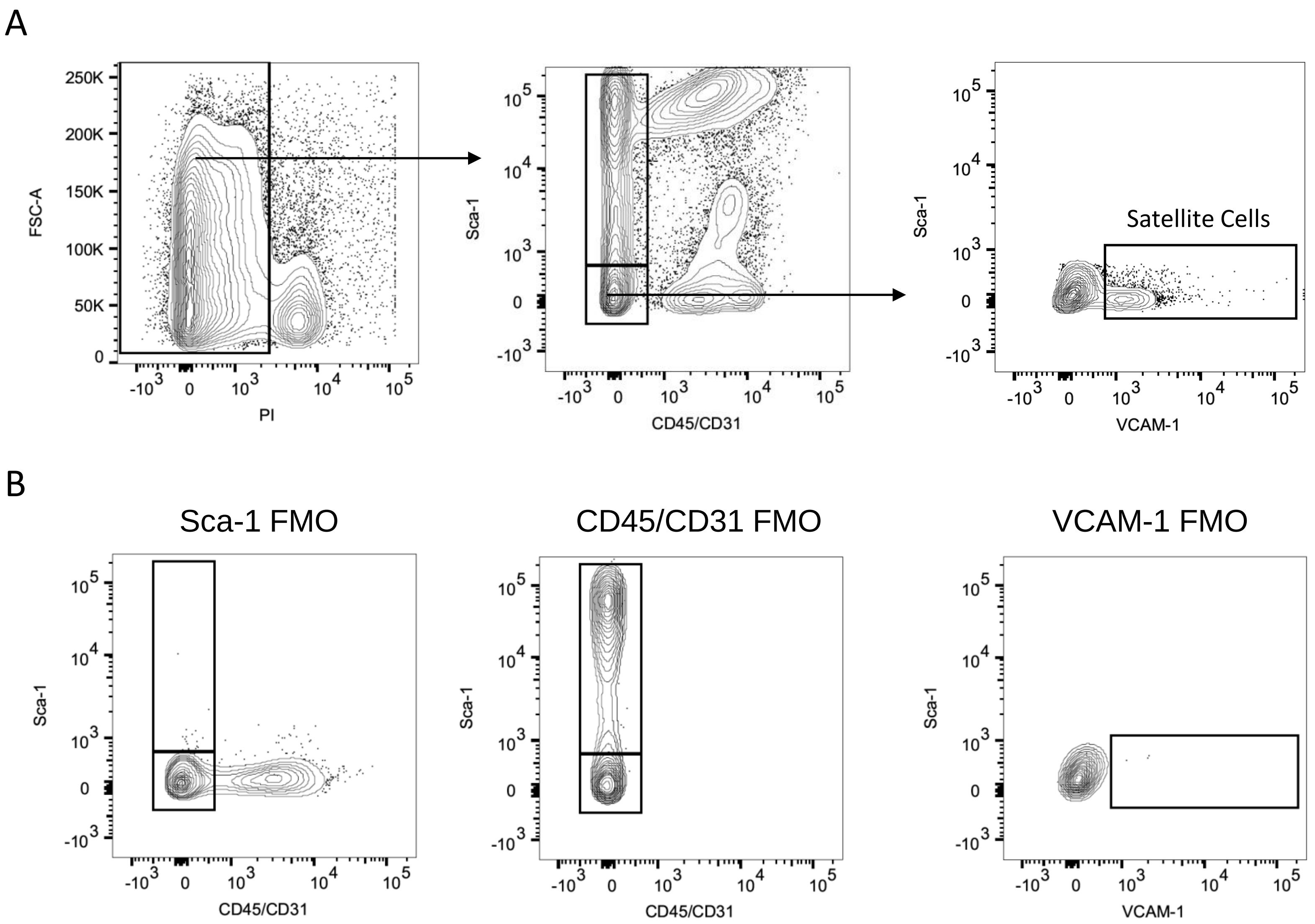


Supplemental Figure S11. Identification of VCAM-1$^{+}$ satellite cells by flow cytometry. A) Representative image depicts isolation of satellite cells in a young uninjured muscle. B) Representative images of (Fluorescence Minus One) FMO controls.

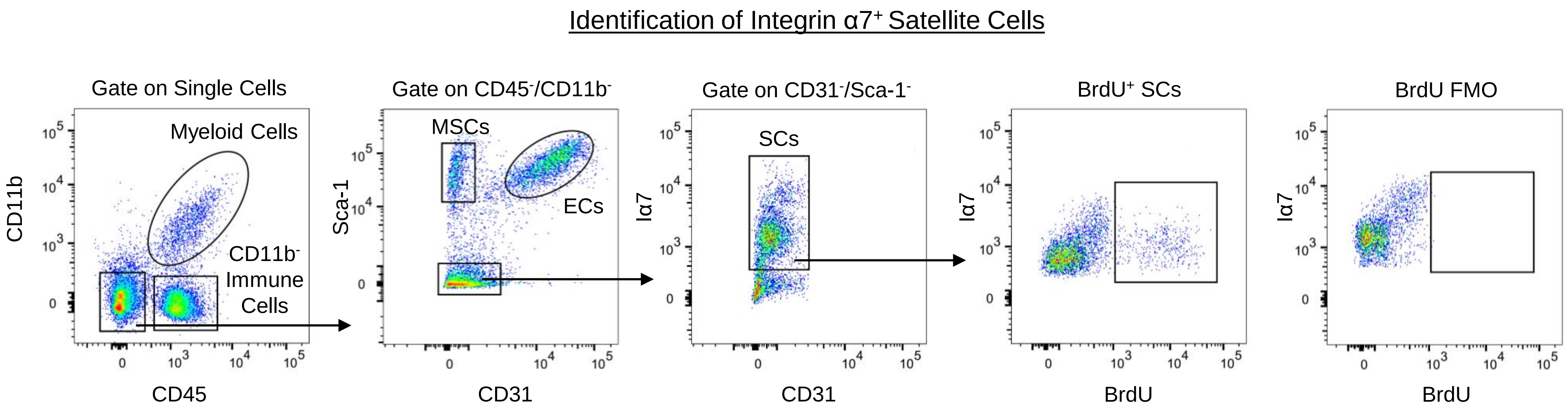


Supplemental Figure S12. Identification of Integrin α7+/BrdU+ satellite cells by flow cytometry. Representative image depicts isolation of satellite cells in young uninjured muscle.

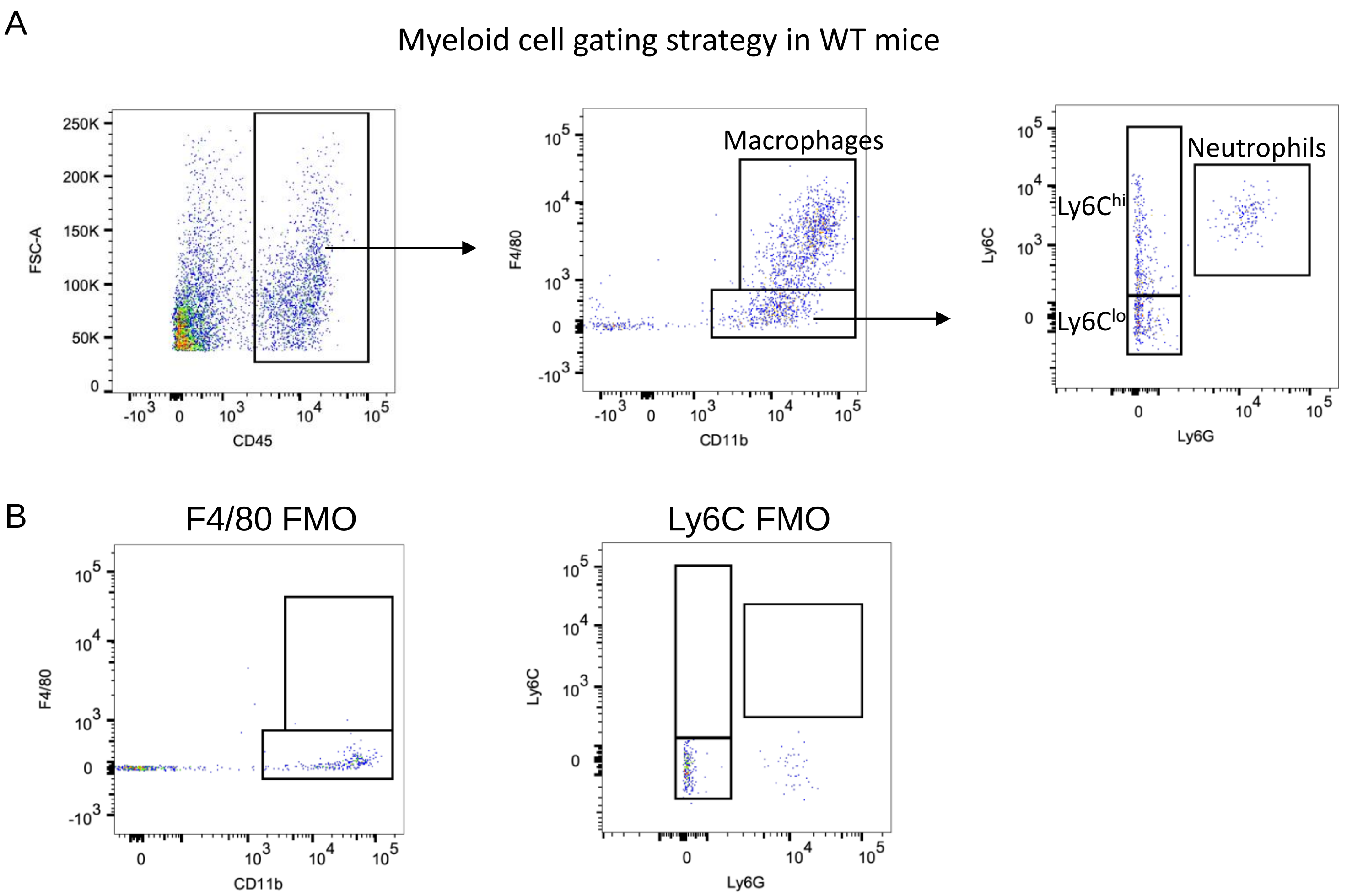


Supplemental Figure S13. Identification of immune cells by flow cytometry. A) Gating strategies to identify CD45 cells, macrophages, neutrophils and monocytes. B) Representative images of (Fluorescence Minus One) FMO controls.

Myeloid cell gating strategy after bone marrow transplant

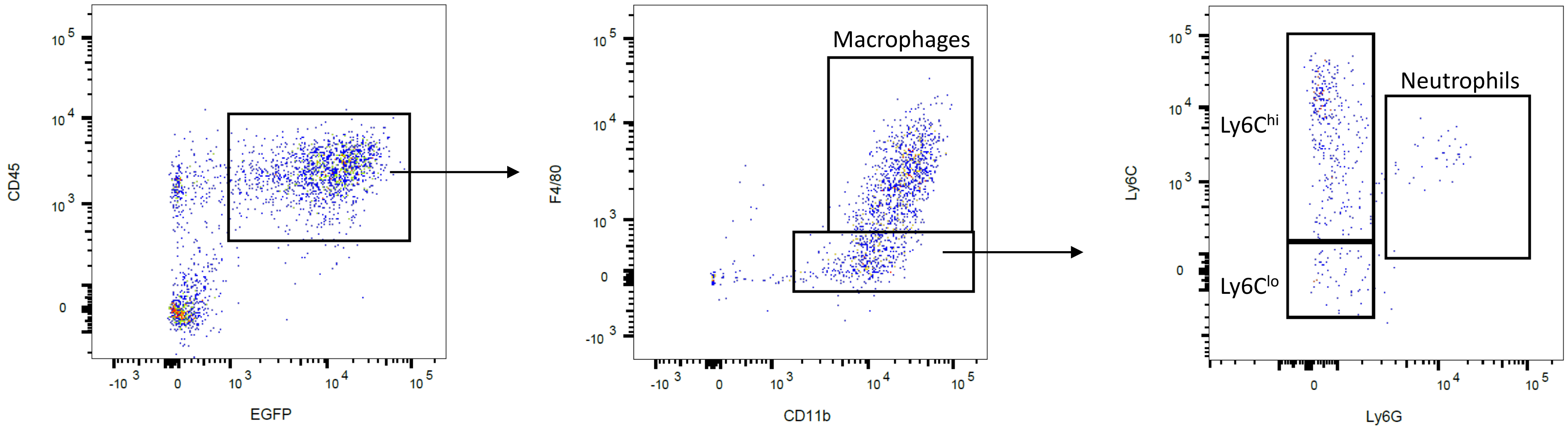


Supplemental Figure S14. Identification of immune cells in mice after bone marrow transplant of $GFP^{+}Sca\text{-}1^{+}$ cells by flow cytometry. Gating strategies to identify CD45 cells, macrophages, neutrophils and monocytes.

T cell Gating Strategy

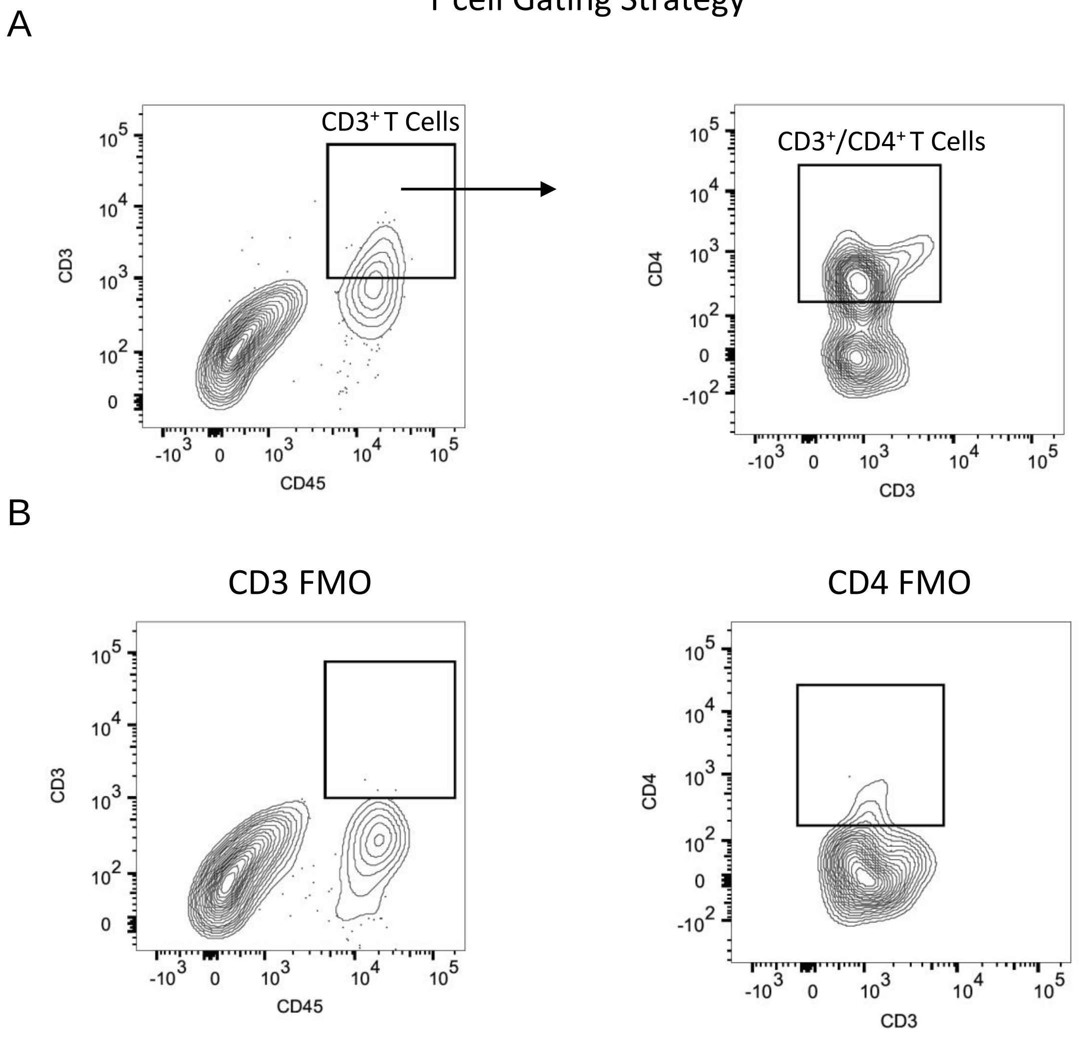


Supplemental Figure S15. Identification of T cells by flow cytometry. A) Gating strategy to identify CD45⁺/CD3⁺/CD4⁺ T cells B) Representative images of (Fluorescence Minus One) FMO controls.
