# Supplementary material for: Delineating the relationship between immune system aging and myogenesis in muscle repair
Source: Aging Cell. 2021 Jan 28;20(2):e13312. doi: 10.1111/acel.13312 (PMC7884032; doi:10.1111/acel.13312)
Supplement: Supplementary file 2 — Supplementary Material [file ACEL-20-e13312-s002.docx]

# Experimental Procedures

***Bone marrow reconstitution*.** The Animal Care Committee of the University Health Network approved all experimental procedures which were carried out according to the Guide for the Care and Use of Laboratory Animals (NIH, revised 2011). Bone marrow from young (3 month) or old (18-20 month) C57BL/6-Tg(CAG-EGFP)1Osb/J enhanced GFP mice was isolated from the tibiae and femur as follows: Cells were dissociated from bone marrow in PBS using an 18G-23G needle. Cells were then incubated in 5 ml of Red Blood Cell lysis buffer (154.42 mM NH_4_Cl, 11.9 mM NaHCO_3_, 0.026 mM EDTA) for 5 min and then centrifuged for 5 min at 1000 rpm. These steps were repeated for a total of two times each. The cell pellet was suspended in PBS and passed through a 40 µm filter to remove debris. Sca-1^+^ cells were purified using Sca-1 magnetic purification (Stem Cell Technology). Old (>18-20 month) C57BL/6J female mice were lethally irradiated at 9.5 Gy and received an infusion (through the tail vein) of 2x10^6^ GFP^+^/Sca-1^+^ cells from young or old GFP mice. Three months later, mice were sacrificed or underwent CTX muscle injury. The reconstitution rate of this model has been previously described (Li et al. 2019).

***Muscle injury and BrdU administration*.** Cardiotoxin was administered as previously described(Guardiola et al. 2017). Briefly, mice were sedated using isoflurane and the hindlimbs were cleaned using ethanol. One, two, or three doses of 20 µl of 10 µM Cardiotoxin (Latoxan, L8102) was injected into the tibialis anterior (TA), quadriceps (Quad), or gastrocnemius (Gastroc) muscles, respectively. The uninjured leg received a volume control injection of Saline into the TA muscle for qRT-PCR analysis. For CTX-BrdU experiments, CTX was administered as described above on day zero. For immunofluorescent staining, 5-bromo-2′-deoxyuridine (BrdU; Sigma-Aldrich) was administered three days after CTX injury via intraperitoneal injection six hours prior to sacrifice at a dose of 50 mg/kg. For flow cytometry experiments, BrdU (50mg/kg) was administered via intraperitoneal injections on days 0, 1 and 2 before termination on day 3.

***Locomotive and behavioural tests.*** Open field test: The open field test apparatus consisted of a 38 x 60 x 60 cm chamber with grey Plexiglas walls and transparent ceiling to allow for video recording. Mice were placed in the chamber for 10 min and their ambulatory distance and rearing count was tracked and recorded. Video files were initially analyzed by idTracker (Pérez-Escudero et al. 2014) in order to obtain x- and y-coordinates associated to activity, followed by subsequent analysis using a custom python script for extraction and calculation of ambulatory distance. The testing apparatus was cleaned with 70% ethanol between each mouse. Rotarod test: The accelerating rotarod test was performed on a Pan Lab Lsi Rota-Rod/RS Model 8200 and the times spent before falling were recorded. Prior to testing, the mice were acclimated to the device at a constant speed (4 rpm) and at constant acceleration (4 rpm to 40 rpm in 30 seconds) for three separate trials for each setting with at least a 10 min interval between each trial. Afterwards, mice were tested on the accelerating rotarod over the course of three separate trials that were averaged to produce the mean amount of time spent on the device before falling. The testing apparatus was cleaned with 70% ethanol between each mouse.

***Flow cytometry.*** Mononuclear cells were isolated from hindlimb muscle for flow cytometry as described (Liu et al. 2015) after perfusion with PBS. For analysis of uninjured muscle, all hindlimb muscle was collected from both hindlegs . To analyze immune cells at three- or ten-days post CTX, cells were isolated from the injured Gastrocnemius and Quadriceps muscles. BrdU analyses of satellite cells, MSCs, and CD11b as well as macrophage polarization (CD206+) was completed using injured TA, quadriceps, and gastrocnemius muscles. To identify BrdU+ cells, after digestion cells were processed and stained according to the manufacturer’s instructions cells (BD Pharmingen BrdU Flow Kit, 559619 and 552598). To determine satellite cells, MSCs and CD11b per mg tissue, cells were assessed in unfixed samples. Isolated mononuclear cells were stained at 4^o^C in FACS buffer (2% FBS in PBS). Antibodies used to detect satellite and mesenchymal stem cells: Sca-1 (Clone E13-161.7, Cat. no. 553336), Biotin anti-mouse CD106 (Clone 429 MVCAM.A, Cat. no. 105703) or Pacific Blue™ anti-mouse CD106 (Clone 429 MVCAM.A, Cat. no. 105722), α7-integrin (Clone 3C12, Cat. no. 130-120-812), CD45 (Clone 30-F11, Cat. no. 103112), CD31 (Clone MEC13.3, Cat. no. 102509), and Streptavidin BV-711 (Cat. no. 405241). Antibodies used to detect Immune Cells: F4/80 (Clone BM8, Cat. no. 123113), Ly6C (Clone HK1.4, Cat. no. 128015), Ly6G (Clone 1A8, Cat. no. 127613), Cd11b (Clone M1/70, Cat. no. 101207), CD45 (Clone 30-F11, Cat. no. 103128 (Alexa Fluor 700) or 103108 (FITC), depending on if samples included GFP signal), CD3 (Clone 17A2, Cat. No. 100205) and CD4 (Clone GK1.5, Cat. No 100413). Antibodies were purchased from BioLegend, BD Biosciences, and Miltenyi Biotec. Neutrophils were identified as CD45^+^ CD11b^+^ F480^-^ Ly6C^+^ Ly6G^+^, monocytes were identified as CD45^+^ CD11b^+^ F480^-^ Ly6C (hi or lo) Ly6G^-^, macrophages were identified as CD45^+^ CD11b^+^ F480^+^, and T cells were identified as CD45^+^ CD3^+^ CD4^+^. Satellite cells were identified based on negative gating for Sca-1, CD45, CD11b and CD31, and positive selection for V-CAM1 or α7-integrin(Liu et al. 2015). Data was acquired on an LSR II (BD Biosciences) flow cytometer and data analyzed using FlowJo software. Representative images of gating strategies used to identify cell populations are shown in Figures S11-S15.

***qRT-PCR.*** Tissue was snap frozen in liquid nitrogen and then ground into a fine powder. RNA was isolated using TRI-Reagent (Sigma) according to the manufacturer’s instructions. cDNA was prepared from 1000ng of RNA using NxGen M-MulV Reverse Transcriptase (Lucigen; 30222-1) according to the manufacturer’s instructions. cDNA expression was analyzed using SensiFAST (Bioline-98005) SybrGreen using the following parameters: 95^o^ 2 min; [95^o^ 5 s; 60^o^ 10 s; 72^o^ 20 s for 40 cycles]. Relative quantification of gene expression was calculated by ∆∆CT method with *Hprt* or *β-actin* as housekeeping genes, where indicated. Experiments were based on an average of two technical replicates. The number of biological replicates used are indicated in the figure legends. Primers are listed in Table S1.

***Enzyme-linked immunosorbent assay (ELISA).*** At three days post-CTX, whole TA muscle was homogenized as followed: Tissue was homogenized into a fine powder over liquid nitrogen. Protein was extracted via sonication (10s pulses x 4) in lysis buffer (50mM Tris (pH 8.0), 150mM NaCl, 1% NP-40, 1mM β-glycerolphosphate, 1mM Na3VO4, 1μg/mL leupeptin, 1μg/mL pepstatin, 1mM phenylmethylsulfonyl fluoride, Sigma-Aldrich). Equal volumes of protein lysate were used for IL-10 or IL-1β protein analysis according to the manufacturer’s instructions (ThermoFisher, Cat # 88-7105-22 and Catalog # 88-7013-22, respectively) which were normalized to total protein concentration, as determine using the DC protein assay (Bio-Rad).

***Immunofluorescence and histology*.** The TA muscle was placed in 2% PFA and incubated in 10%, 20% and finally 30% Sucrose, overnight. Muscle tissue was embedded in OCT and sectioned at 7 µM. Two percent PFA was added to tissue sections for 10 minutes and washed 3X with 1% BSA in PBS. For BrdU analysis, tissue sections were placed in 2M HCl at room temperature for 10 minutes followed by 37^o^C for 20 minutes and washed 3X with 1% BSA-PBS. Next, the tissue was incubated with borate buffer (0.1M Boric acid, pH 8.5) at room temperature for 10 minutes and washed 3X with 1% BSA-PBS. Tissue was permeabilized in 0.5% Triton-X and blocked in 10% BSA for 15 minutes. Antibodies are as follows: Primary antibodies: BrdU (Abcam, ab6326), CD45 (BD, 550539), GFP (ThermoFisher, A21311) and Laminin (Sigma-Aldrich, L9393). Secondary antibodies: Goat anti-Rat Alexa 546 (ThermoFisher, A11081), Chicken anti-Rat, Alexa 647 (ThermoFisher, A-21472) and Donkey-anti-Rabbit (A31573). with 4′,6-diamidino-2-phenylindole (DAPI, Sigma-Aldrich) was used to counterstain nuclei. BrdU^+^/GFP^-^ nuclei were quantified using an average of three fields of view within the injured TA, followed by the average of BrdU^+^/GFP^-^ cells over three independent experiments (biological replicates). For histological analysis, the TA muscle was placed in formalin for 24 hr, washed in 70% ethanol and embedded in paraffin wax. Muscles were sectioned at 7 µm. Hematoxylin and Eosin (H&E) and Masson’s trichrome staining was completed by the pathology lab at University Health Network (Toronto ON). The sections were viewed and photographed using the Olympus Slide Scanner CM-10 (immunofluorescent staining and histology) or the Zeiss LSM700 confocal microscope (immunofluorescent staining), and the digital images were processed with Zeiss ZEN. The cross-sectional area of 60-100 myofibers per sample was calculated using ImageJ software (n=5). Intermuscular fibrosis was quantified based on trichrome staining using ImageJ and normalized to the area selected (5 fields of view, n=3). Central nuclei quantification was determined from H&E staining by counting the number of nuclei present in the center of myofibers using an average of 5 fields of view and normalized to the area selected (n=3).

***Isolation and in vitro culture of peritoneal macrophages.*** Young (3 month) or old (18-20 month) mice were given an intraperitoneal injection with 4% thioglycolate three days before sacrifice, as described previously (Alibhai et al. 2020). On the third day, mice were sacrificed via carbon dioxide inhalation followed by cervical dislocation. Cells were collected by peritoneal lavage and were resuspended in DMEM/F12 media (ThermoFisher) supplemented with 10% FBS and 1% Penicillin-Streptomycin (P/S). Cells were seeded in 12-well plates at 10^6^ cells/ml. The cells were allowed to adhere overnight, and the media was changed the next day before experimentation. LPS was added to cells at a concentration of 10 ng/ml for 4 hr before collection of RNA.

***Treatment of C2C12 myoblasts with conditioned media from peritoneal macrophages.*** C2C12 myoblasts (ATCC) were seeded into a 24-well dish at 2x10^4^ cells/ml in DMEM (Gibco) supplemented with 10% FBS and 1% P/S. The next day, conditioned medium from young or old peritoneal macrophages were added to culture. Conditioned medium from peritoneal macrophages was collected as follows: Cells were seeded into 6-well plates at 10^6^ cells/ml and stimulated with LPS as described above. After macrophage activation, medium was washed twice with PBS and replaced with 1.5 ml serum-free DMEM/F12 media for 24 hr. Conditioned media was collected, supplemented with 10 µM BrdU, and added to proliferating C2C12 myoblasts one day after seeding. Control C2C12 cells received serum-free DMEM+1% P/S. After 24 hrs, C2C12 myoblasts were fixed in 2% PFA (10 minutes). Antigen retrieval was completed as described above, followed by permeabilization in 0.2% Triton-X and 10% BSA for 30 minutes. Antibodies used for *in vitro* immunofluorescent staining: anti-BrdU (1:200; Abcam, ab6326); Goat anti-Rat Alexa 546 (1:400; ThermoFisher, A11081) and DAPI. Cultured media from LPS-stimulated peritoneal macrophages was collected as previously described. At 90% confluency, C2C12 myoblasts were grown in an equal mixture of LPS conditioned media and differentiation media for 4 days. Control cells were cultured in an equal mixture of F-12/DMEM (1% P/S) and differentiation media.

***Treatment of C2C12 myoblasts with recombinant cytokines.*** C2C12 myoblasts were seeded into a 24-well dish at 2x10^4^ cells/ml in DMEM supplemented with 10% FBS and 1% P/S. The next day, BrdU (10 µM) and recombinant IL-1β (201-LB, R&D Systems), IL-12p70 (210-21, PeproTech), TNFα (210-TA, R&D Systems), IL-10 (210-10, PeproTech) or IFNγ (485-MI, R&D Systems) was added to proliferating myoblasts at a final concentration of 10 ng/ml in serum-free DMEM (plus 1% P/S) for 24 hr. For the differentiation and fusion assays, C2C12 myoblasts were grown to 90% confluency before switching to differentiation medium (2% Horse serum (Gibco) plus 1% P/S in DMEM) supplemented with one of the listed cytokines (10 ng/ml) for 4 days. Media and cytokines were refreshed after 72 hr.

***Calculation of proliferation, differentiation, and fusion indices***. To calculate the proliferation index, BrdU^+^ nuclei were quantified on ImageJ and normalized to the total number of nuclei present per field. Nuclei were averaged from two fields per biological replicate (n=3). For the differentiation index, all mono- or multi-nucleated cells expressing myosin (MY-32, M4276, Sigma-Aldrich) were quantified and normalized to the total number of nuclei per field. The fusion index was calculated based on the number of nuclei in myosin expressing myotubes normalized to the total number of nuclei per field. Myotubes were defined as ≥2 nuclei/cell. Nuclei were quantified using Image J.

***Statistics*.** Data are depicted as mean ± SEM. Experiments with two groups were analyzed using a two-tailed unpaired t-test. Experiments with one variable and multiple groups were analyzed using a one-way ANOVA followed by Tukey’s post-hoc test; Experiments with two variables were analyzed using a two-way ANOVA followed by Bonferroni’s post-hoc test (GraphPad Prism 5). Significance was accepted as P < 0.05.

Alibhai F.J., Lim, F., Yeganeh, A., DiStefano, P.V., Binesh-Marvasti, T., Belfiore, A., Wlodarek, L., Gustafson, D., Millar, S., Li, S.H., Weisel, R.D., Fish, J.E. & Li. R.K. (2020). Cellular senescence contributes to age-dependent changes in circulating extracellular vesicle cargo and function. *Aging Cell,* *19*, e13103.

Guardiola, O., Andolfi, G., Tirone, M., Iavarone, F., Brunelli, S. & Minchiotti, G. (2017). Induction of Acute Skeletal Muscle Regeneration by Cardiotoxin Injection. *Journal of Visualised Experiments:Jove*, *119*, e54515–e54515.

Li, J., Li, S.H., Dong, J., Alibhai, F.J., Zhang, C., Shao, Z.B., Song, H.F., He, S., Yin, W.J., Wu, J., Weisel, R.D., Liu, S.M. & Li, R.K. (2019). Long-term repopulation of aged bone marrow stem cells using young Sca-1 cells promotes aged heart rejuvenation. *Aging Cell* *18*, e13026.

Liu, L., Cheung, T.H., Charville, G.W. & Rando, T.A. (2015). Isolation of skeletal muscle stem cells by fluorescence-activated cell sorting. *Nature Protocols, 10*, 1612–1624.

Pérez-Escudero, A., Vicente-Page, J., Hinz, R.C., Arganda, S. & De Polavieja, G.G. (2014). IdTracker: Tracking individuals in a group by automatic identification of unmarked animals. *Nature Methods, 11*, 743–748.
